# Prenatal iron exposure and childhood type 1 diabetes

**DOI:** 10.1038/s41598-018-27391-4

**Published:** 2018-06-13

**Authors:** Ketil Størdal, Harry J. McArdle, Helen Hayes, German Tapia, Marte K. Viken, Nicolai A. Lund-Blix, Margaretha Haugen, Geir Joner, Torild Skrivarhaug, Karl Mårild, Pål R. Njølstad, Merete Eggesbø, Siddhartha Mandal, Christian M. Page, Stephanie J. London, Benedicte A. Lie, Lars C. Stene

**Affiliations:** 1 Department of non-communicable diseases, Norwegian Institute of Public Health, Oslo, Norway; 2 Pediatric Department, Ostfold Hospital Trust, Fredrikstad, Norway; 3 The Rowett Institute of Nutrition and Health, University of Aberdeen, Foresterhill, Aberdeen, Scotland UK; 4 Department of Medical Genetics, University of Oslo, Oslo University Hospital, Ullevål, Oslo, Norway; 5 Department of Immunology, Oslo University Hospital, Rikshospitalet, Oslo, Norway; 6 Department of Environmental Exposure and Epidemiology, Norwegian Institute of Public Health, Oslo, Norway; 7 Department of Paediatric and Adolescent Medicine, Oslo University Hospital, Oslo, Norway; 8 Department of Paediatrics and Adolescent Medicine, Haukeland University Hospital, Bergen, Norway; 9 KG Jebsen Center for Diabetes Research, Department of Clinical Science, University of Bergen, Bergen, Norway; 10 Centre for Biostatistics and Epidemiology, Oslo University Hospital, Oslo, Norway; 11 National Institute of Environmental Health Sciences, National Institutes of Health, Department of Health and Human Services, Research Triangle Park, Durham, NC 27709 USA

## Abstract

Iron overload due to environmental or genetic causes have been associated diabetes. We hypothesized that prenatal iron exposure is associated with higher risk of childhood type 1 diabetes. In the Norwegian Mother and Child cohort study (n = 94,209 pregnancies, n = 373 developed type 1 diabetes) the incidence of type 1 diabetes was higher in children exposed to maternal iron supplementation than unexposed (36.8/100,000/year compared to 28.6/100,000/year, adjusted hazard ratio 1.33, 95%CI: 1.06–1.67). Cord plasma biomarkers of high iron status were non-significantly associated with higher risk of type 1 diabetes (ferritin OR = 1.05 [95%CI: 0.99–1.13] per 50 mg/L increase; soluble transferrin receptor: OR = 0.91 [95%CI: 0.81–1.01] per 0.5 mg/L increase). Maternal but not fetal *HFE* genotypes causing high/intermediate iron stores were associated with offspring diabetes (odds ratio: 1.45, 95%CI: 1.04, 2.02). Maternal anaemia or non-iron dietary supplements did not significantly predict type 1 diabetes. Perinatal iron exposures were not associated with cord blood DNA genome-wide methylation, but fetal *HFE* genotype was associated with differential fetal methylation near *HFE*. Maternal cytokines in mid-pregnancy of the pro-inflammatory M1 pathway differed by maternal iron supplements and *HFE* genotype. Our results suggest that exposure to iron during pregnancy may be a risk factor for type 1 diabetes in the offspring.

## Introduction

The rapid increase in incidence of type 1 diabetes (T1D) over the past 2–3 generations demonstrates the importance of yet unknown environmental factors^1,2^. Elements from the diet and microbial triggers are the two broad categories suspected to contain these environmental factors^1^. A large number of gene variants have been characterized with predisposing or protecting effects, with common variants in the HLA class II genes shown to be responsible for the majority of genetic predisposition^3^.

The recognition of conditions during the fetal period and early life as predictors for later health and disease has led to an immune programming hypothesis, underlining the plasticity of the developing immune system modified by maternal nutrition, micronutrients, and gut microbiota^4–7^. Iron is important for the development and function of the immune system and has been shown to influence adaptive immune responses and macrophage function^8^.

Excess intestinal uptake of iron which occurs in hereditary hemochromatosis may lead to T1D, classically described as bronze diabetes, due to pancreatic iron accumulation^9^. Common variants in the *HFE* gene (p.282Y and p.63D) are associated with cellular uptake of iron and hemochromatosis^10,11^. Both maternal and fetal *HFE* genotype likely influences fetal iron status^12^. The *HFE* variants have been found more frequently among individuals with late-onset T1D^13^.

**Figure 1:**
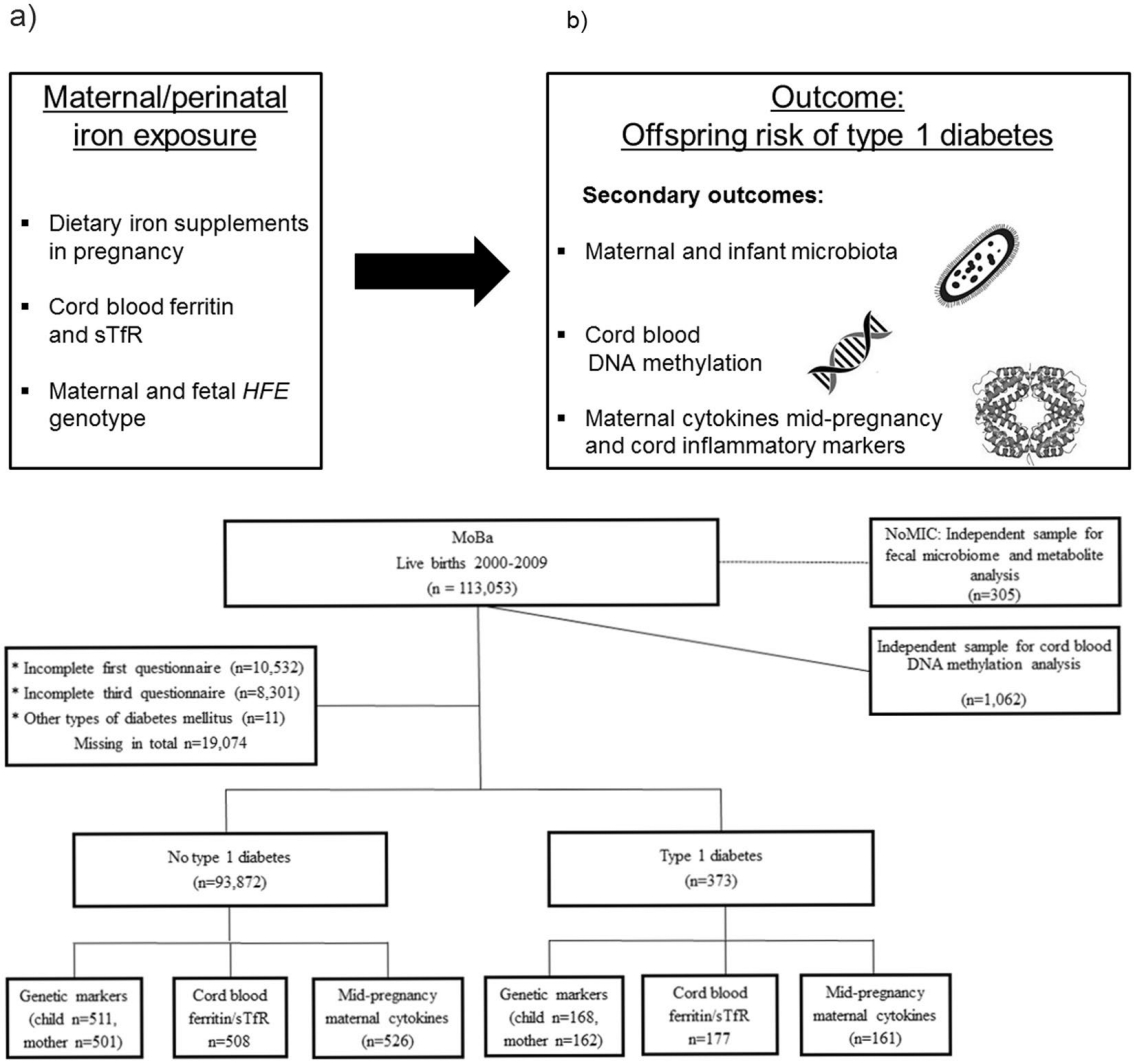
Research questions and study design. (**a**) Illustration of exposures and outcomes (sTfR: soluble transferrin receptor) This figure is not covered by the CC BY licence. [Picture: ^©^ Can Stock Photo/Eraxion, ^©^ Can Stock Photo/adekvat and from http://www.ebi.ac.uk/.]. All rights reserved, used with permission. (**b**) Flow chart illustrating the formation of the study sample in the MoBa cohort, including sub-study of biomarkers. By 1^st^ of January 2013, cases (n = 276) and random controls (n = 1010) were identified for the study of biomarkers, out of whom 177 and 508 respectively had cord blood samples available. The whole cohort was followed up until 1^st^ of May 2017, and 373 developed type 1 diabetes.

In a large prospective study, we tested the hypothesis that iron supplements in pregnancy are associated with offspring risk of T1D. In smaller sub-studies we tested whether *HFE* genetic variants and cord plasma iron biomarkers are risk factors for childhood T1D. In independent studies, we assessed whether prenatal iron exposure also predicted genome-wide fetal DNA methylation, maternal inflammatory cytokines, and gut microbiota, three factors hypothesised to mediate environmental influences on T1D risk (Fig. 1a). Our findings suggest that excess prenatal iron may be a risk factor for T1D.

## Results

Among 94,209 children in the Norwegian Mother and Child Cohort (MoBa) included with complete exposure data from pregnancy, the median time from birth to end of follow-up was 11.5 years (range 7.8–17.3 years). By 1^st^ of May 2017, 373 children (188 girls, 50.4%) had been diagnosed with T1D at a median age of 7.5 years (range 0.7–15.1, Fig. 1b). Participant characteristics are described in Supplementary Table S1.

### Iron supplement use in pregnancy

Iron supplements were used at some point in pregnancy by 64% of the 94,209 pregnancies in the MoBa study, more commonly with increasing gestational age (Supplementary Fig. 1 online). The proportion of users was highest among those with diagnosed anaemia or haemoglobin <10.5 mg/dL at any time during pregnancy, but more than half of those with normal haemoglobin concentrations (lowest haemoglobin ≥12.5 mg/dL) also reported use of iron-containing supplements (Supplementary Table S1). Iron supplement users were less likely to smoke, had higher age and education, lower pre-pregnancy body mass index (BMI) and parity. Maternal *HFE* genotype was not associated with use of iron supplements (Supplementary Table S1).

The median intake of iron from iron-containing dietary supplements among users of supplements was 14.0 mg/d (mean 26.9, SD 39.4 mg/d), whereas the median estimated iron intake from foods at week 17–22 in all pregnancies was 10.7 mg/d (mean 11.2, SD 3.8 mg/d, Supplementary Table S2).

### Maternal iron supplements in pregnancy were associated with higher risk of type 1 diabetes in offspring

**Figure 2:**
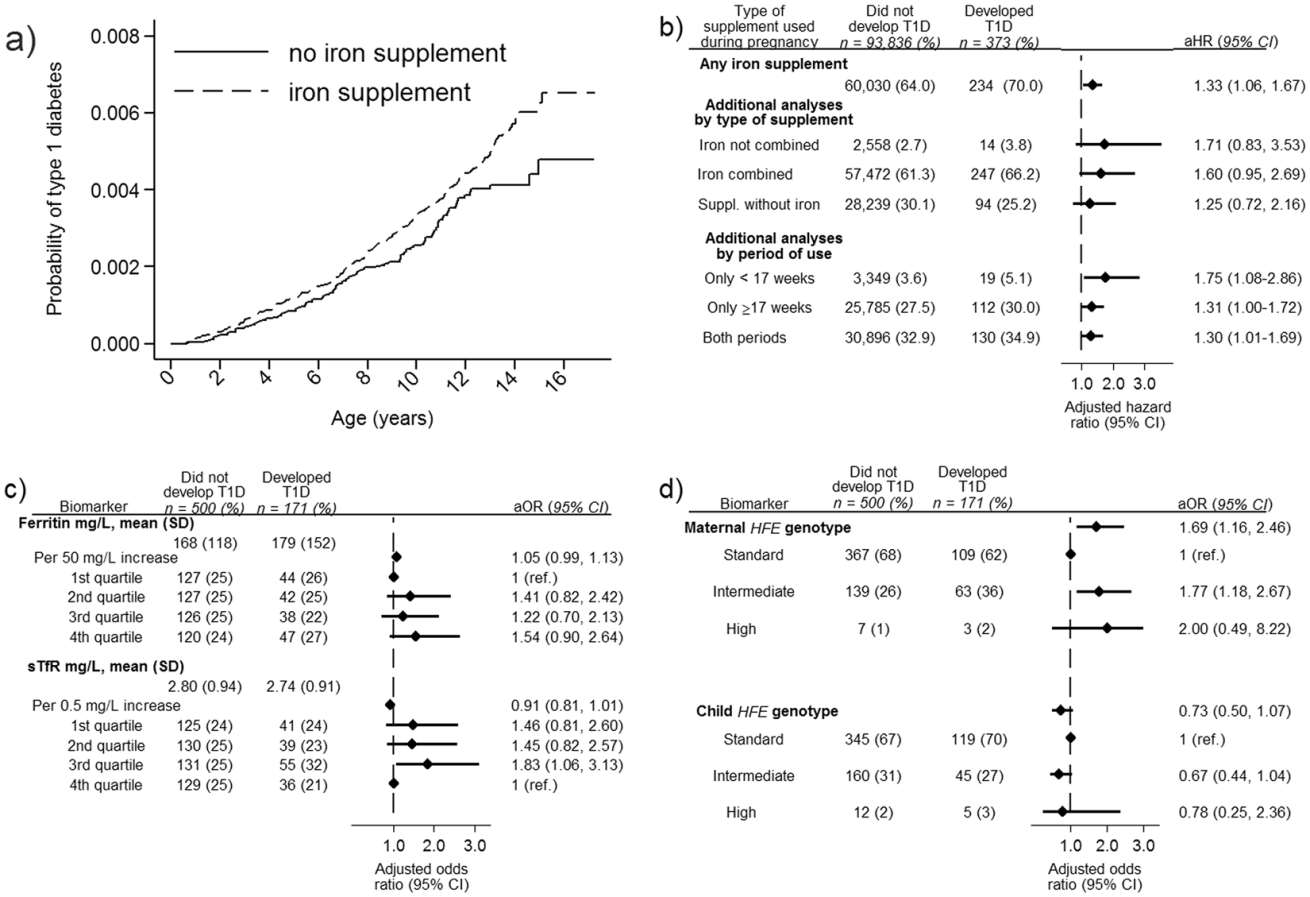
Perinatal iron exposures and risk of type 1 diabetes. (**a**) Risk of type 1 diabetes in the offspring by use of iron-containing supplements during pregnancy. Picture: ^©^Can Stock Photo/Eraxion, ^©^Can Stock Photo/adekvat and from http://www.ebi.ac.uk/. (**b**) Maternal iron supplement use, type of supplement used, duration of iron supplements and risk of type 1 diabetes. (**c**) Cord blood ferritin and soluble transferrin receptor (TfR) among children who later developed type 1 diabetes (cases) and randomly selected controls from the same cohort. (**d**) Maternal and fetal *HFE* genotype among children who later developed type 1 diabetes (cases) and randomly selected controls from the same cohort. *Panel b and c* Adjusted for maternal age and education, smoking, parity, pre-pregnancy BMI, mode of delivery, birth weight and prematurity, maternal type 1 diabetes, maternal celiac disease and diagnosed maternal anaemia (<17 weeks). Panel c additionally adjusted for year of birth. Ferritin quartiles: 1^st^ quartile < 96 mg/L, 2^nd^ quartile 96–142 mg/L, 3^rd^ quartile 142.1–207 mg/L, 4^th^ quartile >207 mg/L. sTFR quartiles: 1^st^ quartile <2.15 mg/L, 2^nd^ quartile 2.15–2.63 mg/L, 3^rd^ quartile 2.631–3.31 mg/L, 4^th^ quartile >3.31 mg/L. *Panel d* Reciprocally adjustment for maternal/fetal genotype Standard genotype: Wild type *HFE* allele at both loci (C282 and H63). Intermediate genotype: p.63D/63D homozygotes and 63D or 282Y heterozygotes High genotype: p.282Y homozygous or p.282Y/63D compound heterozygotes.

The incidence rate of T1D among children whose mothers had used iron supplementation was 36.8/100,000/year compared to 28.6/100,000/year among those without supplementation. The hazard ratio (HR) for T1D was 1.33 (95%CI 1.06–1.67) after adjustments for maternal age and education, smoking, parity, birth weight and prematurity, pre-pregnancy BMI, mode of delivery, diagnosed maternal anaemia, maternal T1D and maternal celiac disease (Fig. 2a).

The risk was consistently increased in children both if their mothers had used iron-only supplements (adjusted HR 1.71, 0.83–3.53), and if their mothers had used iron supplements that also included other nutrients (adjusted HR 1.60, 0.95–2.69, Fig. 2b). This suggests that the observed association was not driven by an underlying reason for taking iron supplements such as anaemia. Offspring whose mothers had used iron supplements only before 17 weeks of gestation had the highest risk (adjusted HR 1.75, 1.08–2.86), with intermediate increased risks for iron supplements only in the second part or in both periods (Fig. 2b).

### Maternal iron from food, anaemia and haemoglobin level were not risk factors for type 1 diabetes

The amount and inter-individual variation of iron intake from foods were typically much lower than that from iron containing supplements. Iron intake from foods during pregnancy was not associated with T1D in the offspring (adjusted HR for highest vs lowest quartile 1.05, 95% CI 0.77–1.42, Supplementary Table S2).

A diagnosis of maternal anaemia did not independently predict T1D, and haemoglobin <10.5 g/dL recorded during pregnancy was not associated with T1D in the offspring (Supplementary Table S2). Adjusting the main analysis for low haemoglobin yielded virtually unchanged risk estimates (HR 1.34, 95% CI 1.04–1.74). Stratifying the analysis by pregnancies with lowest haemoglobin <10.5 g/dL, we found risk estimates similar to those in the full cohort analysis (data not shown). Restricting to pregnancies without a diagnosis of anaemia yielded identical HR estimates to the full cohort analysis (data not shown). Again, this supports the notion that the observed association between maternal iron supplements during pregnancy and T1D was not driven by anaemia as an underlying reason for taking iron supplements.

Of the full cohort, in 10.8% either the mother, the father or both had a non-Norwegian ethnicity. Excluding these from the analysis yielded similar results as the full cohort (data not shown). As expected, HLA-type was a strong predictor for T1D, but the association between iron supplement use and offspring T1D risk was similar across HLA categories (Supplementary Table S3). Perinatal factors (birth weight, gestational age and mode of delivery) could be viewed as mediators, occurring after the main exposure and thus not formally confounding variables. A sensitivity analysis without these covariates yielded risk estimates similar to those in the main analysis (data not shown).

Iron supplements after birth are generally recommended during the first 6–12 months of life in children with gestational age <35 weeks or birth weight <2500 g, and were used by 4.3% and 1.4% at 6 and 18 months age, respectively. Use of iron supplements at 6 or 18 months were not associated with later T1D (Supplementary Table S4).

### Genetic and environmental predictors for biomarkers of iron status in cord plasma

High serum ferritin or low soluble transferrin receptor (sTfR) are biomarkers of high iron stores. We measured these biomarkers in a nested sample of children who developed T1D and randomly selected controls. Maternal iron supplement use at any time during pregnancy or iron intake from foods did not predict cord plasma ferritin among controls (n = 500, Supplementary Table S5). Increasing maternal pre-pregnant BMI showed a suggestive association with lower iron stores, of borderline significance, whereas other maternal factors not associated with iron biomarkers in cord samples (Supplementary Table S5). Ferritin was higher in carriers of the *HFE* high-risk genotype, but these genotypes occurred in only 1.1% of the mothers and 2.3% of the children (Supplementary Table S6).

### Cord plasma iron biomarkers and the risk of type 1 diabetes

Ferritin was slightly higher in cord plasma in individuals who later developed T1D compared to controls (179 vs 168 mg/L). There was a suggestive, but non-significant association between increasing ferritin and risk of childhood onset T1D (adjusted odds ratio 1.05 [0.99–1.13] per 50 mg/L increase, Fig. 2c). Soluble transferrin receptor was slightly lower, indicating higher iron stores, among T1D cases as compared to controls. Again there was a suggestive but non-significant association between lower concentrations and risk of childhood T1D (adjusted odds ratio 0.91 [0.81–1.01] per 0.5 mg/L increase, Fig. 2c).

Ferritin is known to increase during inflammation, and inflammatory markers (neopterin and kynurenine/tryptophan ratio) were weakly positively associated with ferritin and negatively with sTfR (Supplementary Table S7). However, the association of serum ferritin and sTfR with type 1 diabetes was not affected by additional adjustment for these inflammatory markers (aOR 1.06 [0.99–1.13]; aOR 0.92 [0.82–1.02], respectively).

### Maternal HFE variants were associated with offspring type 1 diabetes

Maternal *HFE* genotype was significantly associated with offspring T1D, whereas the child’s *HFE* genotype was not (Fig. 2d).

Since maternal and fetal genotypes are correlated, they may confound each other, so we also ran a model with both included. The association between maternal HFE genotype with T1D was strengthened (OR 1.69, 95%CI 1.16–2.46) whereas the child genotype remained non-significant (Fig. 2d), supporting an effect of maternal but not fetal genotype.

We found the highest point estimate for the maternal genotypes with high risk for hemochromatosis. However, this group was small yielding wide confidence intervals and non-significant results (Fig. 2d).

### Fetal HFE genotype, but not maternal iron supplement use, was associated with differential cord blood DNA methylation (near HFE) at chromosome six

**Figure 3:**
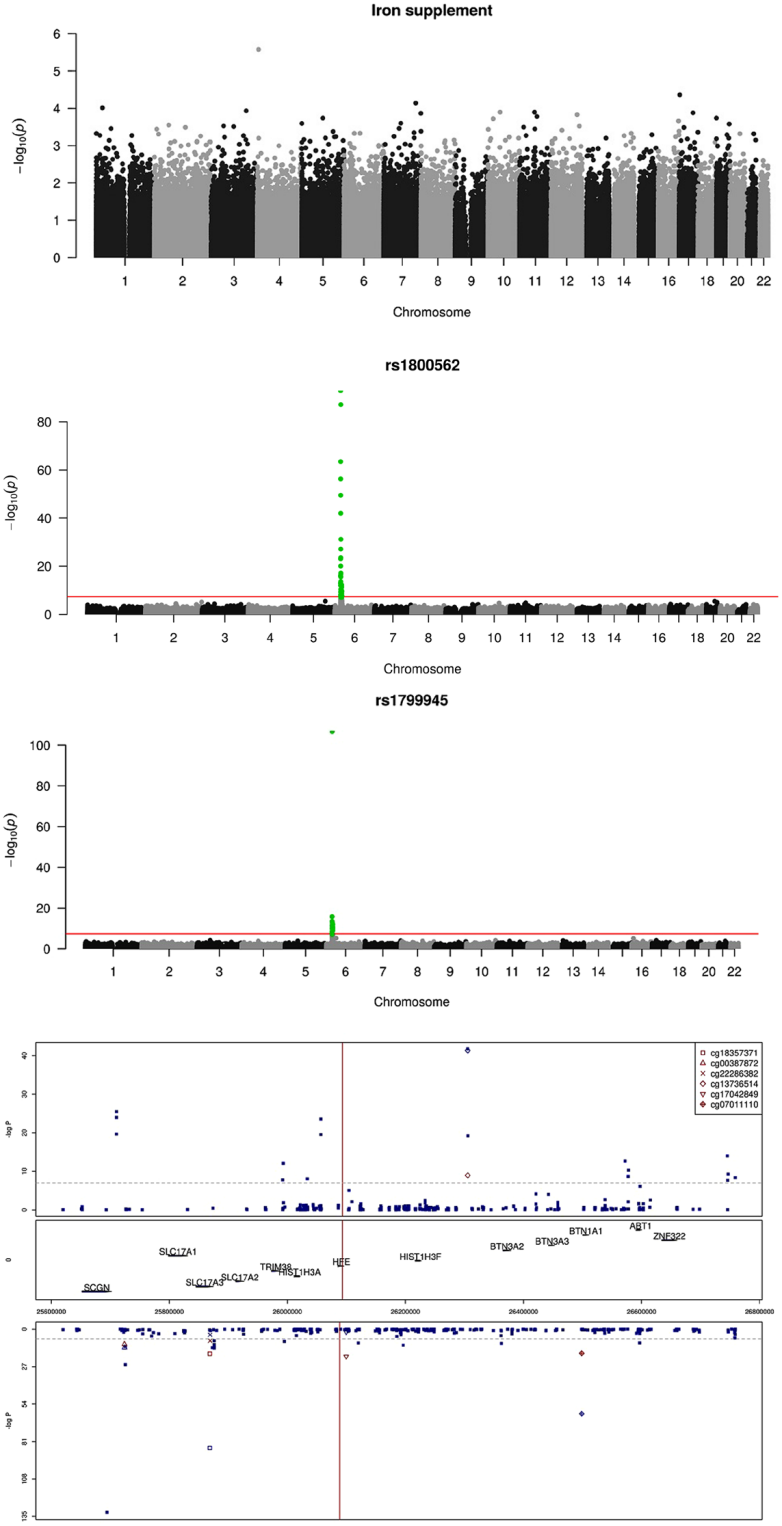
Perinatal iron exposure and genome-wide cord blood DNA methylation. (**a**) No genome-wide significant association between maternal iron supplement use in pregnancy and genome-wide DNA methylation assayed by Illumina 450 K chip, in n = 1,062 mother/child-pairs in the MoBa cohort. (**b**) Methylation in cord blood samples from the MoBa cohort by the coding SNP rs1800562 (p.C282Y) in the *HFE* gene at chromosome 6. (**c**) Methylation in cord blood samples from the MoBa cohort by the coding SNP rs1799945 (p.H63D) in the *HFE* gene at chromosome 6. (**d**) Fetal *HFE* rs1800562 genotype and genome-wide cord blood DNA methylation in n = 1,062 children in the MoBa cohort showed multiple differentially methylated sites near the *HFE* gene with genome-wide significance. rs1800562 is the hemochromatosis associated non-synonymous p.C282Y SNP. The red marks represent associations for the six methylation sites (CpGs) that were replicated in the ALSPAC cohort in the UK, for the same SNP, reported in their metQTL database^16^. The Manhattan plot is split according to direction of association, with positive associations (hypermethylation) associated with the p.C282Y variant) at the top and negative associations (hypomethylation) at the bottom. The mid panel indicates the position of the genes in this region of chromosome six. Numbers on the x-axis indicate genome position.

Since fetal DNA methylation has been proposed to mediate effects of *in utero* environment on later health and disease^14^, we tested whether use of iron supplements or *HFE* genotypes were associated with DNA methylation signatures in cord blood using data from a genome-wide Illumina 450 K methylation chip^15^. In an independent random sample of 1,062 children in the MoBa cohort (Fig. 1b), maternal use of iron supplements during pregnancy was not associated with fetal DNA methylation pattern (Fig. 3a).

However, we found that the fetal p.282Y *HFE* genotype (rs1800562) was strongly associated with methylation at several CpG sites in proximity to the *HFE* gene at chromosome six (Fig. 3b). This finding was replicated for six of these sites in another independent cohort of 1000 newborns in the ALSPAC study^16,17^, Fig. 3d. Likewise, for the other tested *HFE* SNP (rs1799945/p.63D) we also found differential methylation around the SNP (Fig. 3c).

Next, we tested whether there were other SNPs near *HFE* more strongly associated with methylation in the replicated CpG sites. Several other SNPs within + /− 30,000 kbp were also significantly associated with differential methylation at the replicated CpG sites (Supplementary Fig. 2a), most of which were in weak or no linkage disequilibrium with the *HFE* rs1800562 SNP (Supplementary Fig. 2b). This suggest that the differential methylation seen with *HFE* rs1800562 was not due to a technical artefact involving disruption of a CpG site or probe binding site, but also questions the causal role of HFE rs1800562 in methylation.

Given the surprisingly strong association between the hemochromatosis linked *HFE* variant and cord blood DNA methylation, we decided to also test whether established non-HFE iron status SNPs for differential methylation SNPs. All three tested SNPs (for *TF* and *TMPRSS6*) showed genome wide significant differential methylation in cord DNA, generally at sites near the corresponding SNP (Supplementary Fig. 3a–c). Overall, there seems to be strong differential methylation associated with SNPs influencing iron status, but the significance and causal interpretation of these observations are unclear.

### Iron supplement use and cytokines in mid-pregnancy

Systemic inflammation is thought to be involved in a number of chronic diseases, and is tightly linked to iron metabolism^18^. An integrated measure of grouped cytokines linked to the pro-inflammatory type M1 macrophage in maternal samples from mid-pregnancy differed significantly by iron supplementation (Supplementary Fig. 4c).

Use of iron supplements from 0–17 weeks of pregnancy were not significantly associated with any of the 18 individual cytokines in maternal samples in mid-pregnancy (Supplementary Fig. 4a,b).

Maternal *HFE* genotype (rs1800562) was associated with increased concentrations of interferon-γ and CCL4 (Supplementary Fig. 5). Overall, we conclude that there was suggestive evidence from our studies that maternal/fetal iron exposure was associated with increased systemic inflammation.

### Iron supplements, fecal microbiota and metabolites

The intestinal microbiota is thought to influence the risk of T1D, and the composition is influenced by a number of environmental factors such as use of antibiotics and diet^19^. We next studied whether maternal iron supplement was associated with changes in the maternal and infant microbiota. Fecal specimens from term infants sampled during the first year of life and their mothers in the Norwegian Microbiota Study (NoMIC, Fig. 1)^20,21^ were subjected to 16 S ribosomal RNA sequencing. Maternal iron supplements during pregnancy was associated with a suggestive increase in microbial diversity in maternal samples of borderline statistical significance, but there was no significant difference in infant samples (Supplementary Table S8).

Overall, the ratios of major microbial families did not differ by maternal iron supplementation use, except for the ratio of *Bacteroidaceae:Clostridiaceae*, which was markedly higher from 4 months to 2 years (p = 0.008) among infants of mothers who used iron supplements during pregnancy (Supplementary Table S9). The ratio of *Bacteroidaceae:Enterobacteriaceae* was non-significantly increased both during the first month and from 4–24 months.

However, in the maternal faecal samples, the relative abundance of four operational taxonomic units (OTUs) differed by maternal iron supplement belonging to either the *Ruminococcaceae* or *Lachnospiraceae* family in the Clostridial order. In the infants’ fecal samples, four other OTUs (*Enterobacteriaceae* and *Streptococcaceae*), differed according to maternal iron use during pregnancy (Supplementary Fig. 6).

Eight short-chain fatty acids (SCFAs) metabolites produced by gut microbes were measured in infant fecal samples in the NoMIC study using gas chromatography. The overall SCFA composition in infant fecal samples was similar in those exposed to maternal iron and those not. The only observed difference was significantly higher levels of the SCFA caproic acid at day 4 in exposed infants (Supplementary Table S10).

## Discussion

In one of the world’s largest pregnancy cohort, we have described for the first time a prospective association between maternal use of iron supplements in pregnancy and a higher risk of childhood T1D. The observation that a maternal genetic variant in *HFE* affecting intestinal absorption of iron and possibly trans-placental transport of iron, adds credibility to this observation. Our independent studies of the association between prenatal iron exposures and factors thought to mediate environmental influences on the risk of type 1 diabetes, suggested that these factors may have mediated the association, although the latter was admittedly not conclusive.

The size of the MoBa study makes it suitable to study aetiological factors including gene-environment interactions in diseases with relatively low prevalence. Another important strength is the fact that questionnaires were completed during pregnancy, which minimized the recall time and risk of recall bias for the exposure of interest. Further, the study is population-based and origins from a relatively homogenous population^22^. In some populations, iron supplements are used by the vast majority of pregnant mothers, reducing the possibility to study prenatal iron as an exposure^23^.

We assessed multiple potential confounders, and the association was robust to adjustments. Use of non-iron supplements were not associated with T1D, which suggests that the association was not confounded by health consciousness or other behavioural factors related to use of dietary supplements. The possibility of association by indication, i.e. that the cause of iron supplement use is the main risk factor, was addressed in a series of analyses showing that this is not a likely explanation of our observation.

The study has some limitations. As in any observational study, causal relationships cannot be established. Despite the adjustment for potential confounders in this large dataset, residual confounding may have influenced our estimates. Limited power for some of the sub-analyses (for example infrequent genotypes) is another weakness which may have given rise to chance findings.

Dietary iron has a number of biological effects, and both low and high intakes can be detrimental to health. A point illustrating the potential public health relevance of our findings is the large international variation in recommendations for dietary iron in pregnant women^23–25^. For the first half of the recruitment period (2000–2005), national guidelines in Norway recommended to measure serum ferritin and to prescribe iron supplements for those with low levels. Revised guidelines did not recommend serum ferritin measurements, but rather recommended haemoglobin measurement at week 30. This is less sensitive to moderate iron deficiency, and likely explains the lower proportion of use in the latter recruitment period^26^. The proportion using iron supplements was high even for those with the lowest reported haemoglobin >12.5 g/dL, which suggests that iron supplementation was given as a broad recommendation during the entire study period and only to a minor degree guided by biomarkers or due to anaemia.

Iron intake from foods was not associated with T1D risk. We do not regard this as a contradiction to the main findings, because the range of variation in dietary intake is much lower than the doses given as supplements; the median total daily iron intake among users of iron supplements was 2.5 times higher than among non-users. Iron supplementation of the child was uncommon, and is recommended only in selected subgroups with lower body iron. Thus, our study probably had insufficient statistical power to study a potential association between the child’s use of iron supplements and risk of T1D.

Biomarkers of increased iron stores have been reported to be increased in patients with T1D^27^, and associated with increased risk of T1D in population-based cohorts^28^. In accordance with our observations, a recent case-control study in Denmark found that iron measured from neonatal dried blood spots was positively associated with childhood-onset T1D^29^. Given the limitations of serum iron, our study provides a more accurate reflection of iron status.

Interestingly, *HFE* variants have also been associated with increased risk of adult onset T1D^13^. However, none of these studies assessed maternal iron supplementation or *HFE* genotypes.

While we have not done any direct experimental or mechanistic studies, we tested in independent studies whether perinatal iron exposures were associated with three potential mediators of our observed link between maternal iron supplement use and childhood T1D: maternal systemic inflammation^30^, fetal DNA methylation^31,32^ and fecal microbiota and SCFAs^33^. Briefly, maternal cytokines of the pro-inflammatory M1 pathway were increased with iron supplement use, and fetal alleles associated with increased iron levels were associated with differential methylation in proximity to the encoding regions. Here, we speculate on potential mechanisms and how these relate to our observations.

Iron plays an essential role as a cofactor for fuel oxidation and electron transport, but it also has the potential to cause oxidative damage if not carefully regulated^34^. Iron is required for normal β-cell function, whereas the β-cell is extremely vulnerable to oxidative stress^35^. The β-cell accumulates iron during pro-inflammatory cytokine attack via upregulated iron absorption, which can lead to formation of reactive oxygen species and β-cell apoptosis^36^. In an animal model, iron supplementation to rats with streptozotocin-induced diabetes resulted in increased pancreatic inflammation and oxidative damage^37^. Markers of oxidative stress in placental tissues have been found after iron supplementation during pregnancy in humans^38^, but adverse clinical effect on the offspring has, to our knowledge, not been reported.

Haematological diseases requiring frequent transfusion and subsequently iron overload lead to an increased risk of diabetes. Pre-diabetes and diabetes in patients with thalassemia has demonstrated that both decreased insulin secretion and resistance may be involved^39–41^. In early stages of transfusion-related diabetes insulin secretion defects tend to be most prominent^42,43^.

Our observations with disease onset years after the exposure to maternal iron supplements may suggest programming effects on the immune system. Macrophages have a central role in iron metabolism, and accumulation of iron in macrophages has been demonstrated to activate these into a pro-inflammatory M1 phenotype, with a putative role of iron in chronic inflammatory and autoimmune conditions^44^. In our study, individual M1 cytokines did not differ significantly by maternal iron supplement use. However, combined M1 cytokines in an integrated measure among mothers in mid-pregnancy differed by use of iron supplements. Furthermore, maternal *HFE* genotypes were significantly associated with two T-helper lymphocyte 1 or M1 macrophage associated cytokines, namely interferon-γ and CCL4, in the maternal plasma mid-gestation. In a recent study from our group using the same study sample, we found that cytokines related to activated M1 macrophages may be associated with later risk of T1D in the offspring^45^. Thus, we may speculate that macrophage polarization may differ in the fetus by iron exposure, although additional, experimental studies are required to support this hypothesis.

Maternal iron intake has been linked to metabolic disease through increased systolic blood pressure in the offspring, possibly through epigenetic mechanisms^46^. In our study, fetal DNA methylation in proximity to the *HFE* gene differed by fetal *HFE* genotype but not by maternal iron supplement use. None of the differentially methylated sites have previously been linked to T1D, and although our findings were among the most significant reported in epigenome-wide association studies to date, the interpretation of these findings requires further study.

The role of the human gut microbiome in development of diabetes in T1D remains unclear^47^. In mice, the maternal gut microbiota has been shown to influence postnatal immune development and diabetes in the offspring^48,49^, and microbial metabolites to protect against T1D^33,50^. Interestingly, a recent study found support for an increase in *Bacteroides* species compared to *E.coli* in cohorts with different incidence of T1D^19^. This ratio was non-significantly increased in our cohort among children exposed to iron, whereas *Bacteroidaceae:Clostridiaceae* was increased from 4 months to two years.

Iron excess can change the gut microbiome and favour multiplication of bacteria dependent on iron (ie, *Enterobacteriaceae*) at the expense of iron-independent bacteria (ie, *Lactobacillae*)^51^. Iron supplementation led to decreased abundances of *Faecalibacterium prausnitzii* and *Ruminococcus* spp., among others, in patients with inflammatory bowel disease^52^. The effect of iron supplements on gut flora in healthy, non-anaemic pregnant women and their offspring is, to our knowledge, unknown.

Although our findings of differences in relative abundance of *Ruminococcaceae* spp. with iron supplement use replicates a recent study^53^, our observations do not suggest that iron supplementation is an important predictor of maternal or infant microbiota or metabolite production.

In conclusion, our results suggest that prenatal iron exposure may increase the risk of T1D. Confirmation of causality and potential mechanisms need further replication and experimental studies. The findings may give new clues to the understanding of T1D aetiology with potential implications for future prevention.

## Methods

### Study population and design

We used information from the population-based MoBa study^22^. Participating mothers were recruited across Norway during mid-pregnancy from 1999–2008, gave written informed consent, and their offspring were followed for T1D to 1^st^ of May 2017. The establishment and data collection in MoBa were approved by the Norwegian Data Inspectorate and the Regional Committee for Medical Research Ethics, and the methods used were in accordance with accepted guidelines. The current study is based on MoBa version VIII of the quality-assured data files released for research in February 2014.

Children whose mothers returned the questionnaires regarding use of iron supplements during pregnancy were eligible (n = 94,209, questionnaires available at www.fhi.no/moba). From the full MoBa cohort, we studied a case-control sample with the use of biomarkers. Additionally, random independent samples were studied for DNA methylation and fecal microbiota. Overview of included participants is provided in Fig. 1b, and characteristics of participants in Supplementary Table S1.

In the MoBa study, maternal blood samples were collected in EDTA tubes at approximately 17 weeks of pregnancy, separated at the hospital laboratories and shipped to the biobank. Immediately after birth, an umbilical cord blood sample was collected, shipped to the biobank and plasma separated upon arrival. All samples were stored at −80 °C until analysis^54^. DNA was extracted and stored at the biobank at −20 °C.

### Outcomes: Case definition of clinical T1D and control selection

We used time from birth to clinical diagnosis of T1D as the outcome. Data on T1D in MoBa was obtained from the repeatedly updated nationwide Norwegian Childhood Diabetes Registry (NCDR)^55^ and the Norwegian Patient Register (NPR). Rare cases of monogenic diabetes and type 2 diabetes were excluded^56^. We identified six additional participants with T1D through NPR who were not registered in the NCDR.

For the nested case-control study, we identified participants with a diagnosis of T1D by January 1^st^ 2014 and available biobanked samples (n = 177) and randomly selected controls (n = 517) from the complete cohort for analysis of iron biomarkers in cord plasma (Fig. 1b).

### Exposure: Iron supplements during pregnancy

Participating mothers completed a mailed questionnaire at enrolment around week 15–17 of pregnancy, including detailed information on the use of dietary supplements by month since start of pregnancy and questions regarding diagnosis and treatment of anaemia. Participants completed a similar questionnaire with monthly intervals for supplement use at gestational week 30. At week 22 a detailed food frequency questionnaire (FFQ) was completed, including supplement use with brand name, the frequency and amount allowing for calculation of daily dose at that time point.

For the main analysis, we coded iron supplement use as a binary exposure variable with any supplement containing iron. Iron supplementation was subsequently divided into separate iron supplement or in combination with other nutrients, vitamins and minerals. For timing and duration of supplementation we defined iron supplement use ≤17 weeks of pregnancy, >17 weeks of pregnancy or both periods vs no use. To study a potential dose/response relation, the daily supplement dose was extracted from the questionnaire at week 22 of pregnancy. We estimated the cumulative iron supplement dose from the daily dose and overall duration of use. For those starting iron supplementation after 22 weeks, the median value for daily supplement dose among all users was used as an approximation. Based on the FFQ completed around week 22 of pregnancy, we assessed supplement intake of iron as a continuous variable, and additionally calculated quartiles for the daily intake.

We calculated the intake of iron from foods from the FFQ from the beginning of pregnancy until the date of completion^57^. A validity study in 119 participants from MoBa showed that the correlation between iron intake through foods according to the FFQ used in the whole cohort and that estimated using a 4-day weighed food diary as a reference was good at 0.42 (95% confidence interval [CI] 0.26, 0.56) after energy adjustment^58^.

### Exposure: Cord blood ferritin and soluble transferrin receptor (sTfR)

Serum ferritin and sTfR concentrations were quantified in cord blood plasma at the Rowett Institute of Nutrition and Health, Aberdeen, Scotland using a commercial ELISA (details in Online Methods).

Haemolysis of samples were visually graded by two independent researchers. Ferritin and sTfR were influenced by marked haemolysis occurring in some of our cord plasma samples, and we consequently excluded strongly haemolysed samples from further analyses. To account for the potential impact of inflammation on ferritin and sTfR, we assessed inflammatory markers in the respective cord blood samples (neopterin and kynurenine/tryptophan ratio) as described elsewhere^59^.

### Exposure: HFE genotypes

The Core Facility for Genotyping at Oslo University Hospital performed SNP genotyping using a Custom GoldenGate assay (Illumina, San Diego, CA, US) per manufacturer’s protocol. DNA extraction, genotyping methods and quality control procedures are described in the online supplement^60^. We used tagSNPs (n = 144) and HLA*IMP:02 to impute the main human leukocyte antigen (HLA) class II genotypes and haplotypes conferring a high risk for T1D^61^. The imputed HLA genotype was confirmed using classical HLA genotyping with sequence-specific primers at the University of Bristol, UK^62^. Based on the previously established risk of T1D conferred by HLA genotype, we categorized genotypes into four risk groups (Supplementary Table S3).

We genotyped two coding variants in the *HFE* gene, rs1800562 (p.282Y) and rs1799945 (p.63D) associated with high iron stores^63,64^. We categorised *HFE* genotypes into three main categories based on the two SNPs; associated with high or intermediate iron stores versus neutral (Fig. 2d and Supplementary Table S6).

### Secondary outcome: Cytokines during mid-pregnancy and inflammatory markers in cord blood

We measured 18 cytokines in maternal mid-pregnancy plasma using Bio-Plex protein array systems (Bio-Rad, Hercules, CA), based on xMAP technology (Luminex, Austin, TX) as previously described^59^. Groups of cytokines the M1 pathway were added in a composite z-score. For measuring neopterin, tryptophan and kynurenine we used a high-throughput liquid chromatography tandem mass-spectrometry at the Bevital laboratory (Bergen, Norway)^65^, details in Online Supplement. The kynurenine/tryptophan ratio was calculated by dividing the plasma concentration of kynurenine (nmol/L) by the concentration of tryptophan (µmol/L).

### Secondary outcome: DNA methylation in cord blood

We analyzed DNA methylation in cord blood from 1,062 participants in an independent but partly overlapping sample from MoBa (Fig. 1b). We measured methylation at 485,577 CpGs using Illumina’s Infinium HumanMethylation450 BeadChip^66^. For this subsample with methylation data, we used Illumina HumanCoreExome for genotyping (details in Online Supplement and in^15^).

### Secondary outcome: Stool sample analyses for microbiota and metabolites

The microbiome analysis were performed on data obtained from the NoMIC cohort (https://www.fhi.no/en/studies/nomic), an independent study of 552 children partially overlapping MoBa. We studied fecal samples collected at 4 days from both mother and term infants and thereafter only from the child at day 10 and 30, and at 4, 12 and 24 months^21^.

DNA was extracted from the samples, and subjected to barcoded sequencing of the V4 region of the 16 S rRNA gene using an Illumina HiSeq. 2000 machine^67^.

Two hundred and fifty nine infants had samples available for chemical analyses of eight short chain fatty acids (SCFA). SCFAs were analyzed by gas chromatography in two different laboratories (details in Online Supplement and in^68^).

### Other variables

Based on previous literature we preselected variables that might influence use of iron supplements, concentrations of iron biomarkers and the risk of T1D as potential confounders. Maternal age and parity, sex, year of birth, birth weight, gestational age and mode of delivery were obtained from the Medical Birth Registry of Norway and categorised as indicated in Supplementary Table S1. From the pregnancy questionnaires, we extracted information regarding smoking status and duration of completed or ongoing maternal education, pre-pregnancy BMI as well as a diagnosis and treatment of anaemia during pregnancy. The lowest recorded haemoglobin measurement result was transferred from the maternity record by participants to the questionnaire at 30 weeks.

Parental T1D and celiac disease were ascertained from the first questionnaire (maternal history) and by register linkage to the NPR. For the secondary analyses, questionnaires completed at age 6 and 18 months contained information on child’s use of iron supplements.

### Statistical Methods

We used Cox proportional hazard regression analysis to examine the association of maternal iron supplement use with diagnosis of T1D, reporting HRs with 95% confidence intervals with robust cluster variance estimation to correct for within-family correlation. Follow-up time was counted from date of birth to the first date of T1D diagnosis, death, emigration, or to end of follow-up (May 1st, 2017) whatever came first. We defined statistical significance as 95% confidence intervals for the HR not including 1.00.

We used logistic regression of the nested case-control data to study the association of biomarkers of iron stores and *HFE* variants with T1D. For missing covariates we used chained equations multiple imputation with 25 imputed sets.

In sensitivity analyses, we performed the main analysis in subjects with complete data on all variables. Additionally, we restricted the analysis to pregnancies without anaemia and to families with Norwegian ethnicity. Finally, we stratified the analyses by four categories of HLA risk to assess potential heterogeneity of associations.

### Data availability

The data that support the findings of this study are available from dataaccess@fhi.no but restrictions apply to the availability of these data, which were used under license for the current study, and so are not publicly available. Data are however available from the authors upon reasonable request and with permission of dataaccess@fhi.

## Electronic supplementary material

Supplementary Information

## References

[1] Atkinson MA, Eisenbarth GS, Michels AW (2014). Type 1 diabetes. Lancet.

[2] Rewers M, Ludvigsson J (2016). Environmental risk factors for type 1 diabetes. Lancet.

[3] Concannon P, Rich SS, Nepom GT (2009). Genetics of type 1A diabetes. N Engl J Med.

[4] Calder PC (2006). Early nutrition and immunity - progress and perspectives. Br J Nutr.

[5] Palmer AC (2011). Nutritionally mediated programming of the developing immune system. Adv Nutr.

[6] Rautava S, Luoto R, Salminen S, Isolauri E (2012). Microbial contact during pregnancy, intestinal colonization and human disease. Nat Rev Gastroenterol Hepatol.

[7] Stene LC, Gale EA (2013). The prenatal environment and type 1 diabetes. Diabetologia.

[8] Cherayil, B. J., Ellenbogen, S. & Shanmugam, N. N. Iron and intestinal immunity. *Curr. Opin. Gastroentero*l 27, 523–528, 10.1097/MOG.0b013e32834a4cd1 (2011). 10.1097/MOG.0b013e32834a4cd1 PMC3734539 21785351

[9] Utzschneider KM, Kowdley KV (2010). Hereditary hemochromatosis and diabetes mellitus: implications for clinical practice. Nat Rev Endocrinol.

[10] Allen KJ (2008). Iron-overload-related disease in HFE hereditary hemochromatosis. N Engl J Med.

[11] Thorstensen K, Kvitland MA, Irgens WO, Hveem K, Asberg A (2010). Screening for C282Y homozygosity in a Norwegian population (HUNT2): The sensitivity and specificity of transferrin saturation. Scand J Clin Lab Invest.

[12] Balesaria S (2012). Fetal iron levels are regulated by maternal and fetal Hfe genotype and dietary iron. Haematologica.

[13] Ellervik C (2001). Prevalence of hereditary haemochromatosis in late-onset type 1 diabetes mellitus: a retrospective study. Lancet.

[14] Claycombe KJ, Brissette CA, Ghribi O (2015). Epigenetics of inflammation, maternal infection, and nutrition. J Nutr.

[15] Joubert BR (2012). 450K epigenome-wide scan identifies differential DNA methylation in newborns related to maternal smoking during pregnancy. Environ Health Perspect.

[16] Online database: ARIES: Accessible Resource for Integrated Epigenomics Studies. http://www.ariesepigenomics.org.uk/. Accessed 13.09.2017.

[17] Gaunt TR (2016). Systematic identification of genetic influences on methylation across the human life course. Genome Biol.

[18] Ganz T, Nemeth E (2015). Iron homeostasis in host defence and inflammation. Nat Rev Immunol.

[19] Vatanen T (2016). Variation in Microbiome LPS Immunogenicity Contributes to Autoimmunity in Humans. Cell.

[20] White RA (2013). Novel developmental analyses identify longitudinal patterns of early gut microbiota that affect infant growth. PLoS Comput Biol.

[21] Mandal S (2016). Fat and vitamin intakes during pregnancy have stronger relations with a pro-inflammatory maternal microbiota than does carbohydrate intake. Microbiome.

[22] Magnus P (2016). Cohort Profile Update: The Norwegian Mother and Child Cohort Study (MoBa). International journal of epidemiology.

[23] Yang J (2017). Maternal use of dietary supplements during pregnancy is not associated with coeliac disease in the offspring: The Environmental Determinants of Diabetes in the Young (TEDDY) study. Br J Nutr.

[24] Nordic Nutrition Recommendations 2004, edition 4., (Copenhagen, 2004).

[25] S*creening for Iron Deficiency Anemia in Childhood and Pregnancy: Update of the 1996 U.S. Preventive Task Force Review*. (2006). 20722137

[26] Retningslinjer for svangerskapsomsorgen., (Sosial- og helsedirektoratet, Oslo, 2005).

[27] Thomas MC, MacIsaac RJ, Tsalamandris C, Jerums G (2004). Elevated iron indices in patients with diabetes. Diabet Med.

[28] Ellervik C (2011). Elevated transferrin saturation and risk of diabetes: three population-based studies. Diabetes Care.

[29] Kyvsgaard, J. N. *et al*. High Neonatal Blood Iron Content Is Associated with the Risk of Childhood Type 1 Diabetes Mellitus. *Nutrients* 9, 10.3390/nu9111221 (2017). 10.3390/nu9111221 PMC5707693 29113123

[30] Lindehammer SR (2011). Early-pregnancy cytokines in mothers to children developing multiple, persistent islet autoantibodies, type 1 diabetes, or both before 7 years of age. Am J Reprod Immunol.

[31] Jerram ST, Dang MN, Leslie RD (2017). The Role of Epigenetics in Type 1 Diabetes. Curr Diab Rep.

[32] Paul DS (2016). Increased DNA methylation variability in type 1 diabetes across three immune effector cell types. Nat Commun.

[33] Marino E (2017). Gut microbial metabolites limit the frequency of autoimmune T cells and protect against type 1 diabetes. Nat Immunol.

[34] Hansen JB, Moen IW, Mandrup-Poulsen T (2014). Iron: the hard player in diabetes pathophysiology. Acta Physiol (Oxf).

[35] Lenzen S (2008). Oxidative stress: the vulnerable beta-cell. Biochem Soc Trans.

[36] Hansen JB (2012). Divalent metal transporter 1 regulates iron-mediated ROS and pancreatic beta cell fate in response to cytokines. Cell Metab.

[37] Sampaio AF (2014). Iron toxicity mediated by oxidative stress enhances tissue damage in an animal model of diabetes. Biometals.

[38] Devrim E, Tarhan I, Erguder IB, Durak I (2006). Oxidant/antioxidant status of placenta, blood, and cord blood samples from pregnant women supplemented with iron. J Soc Gynecol Investig.

[39] Dmochowski K, Finegood DT, Francombe W, Tyler B, Zinman B (1993). Factors determining glucose tolerance in patients with thalassemia major. J Clin Endocrinol Metab.

[40] Messina MF (2002). Three-year prospective evaluation of glucose tolerance, beta-cell function and peripheral insulin sensitivity in non-diabetic patients with thalassemia major. J Endocrinol Invest.

[41] Merkel PA (1988). Insulin resistance and hyperinsulinemia in patients with thalassemia major treated by hypertransfusion. N Engl J Med.

[42] Jaruratanasirikul S (2008). Prevalence of impaired glucose metabolism in beta-thalassemic children receiving hypertransfusions with a suboptimal dosage of iron-chelating therapy. Eur J Pediatr.

[43] Simcox JA, McClain DA (2013). Iron and diabetes risk. Cell Metab.

[44] Recalcati S, Locati M, Gammella E, Invernizzi P, Cairo G (2012). Iron levels in polarized macrophages: regulation of immunity and autoimmunity. Autoimmun Rev.

[45] Vistnes, M. *et al*. Plasma immunological markers in pregnancy and cord blood: A possible link between macrophage chemoattractants and risk of childhood type 1 diabetes. *American Journal of Reproductive Immunology (accepted)* (2017). 10.1111/aji.12802 29266506

[46] Alwan NA, Cade JE, Greenwood DC, Deanfield J, Lawlor DA (2014). Associations of maternal iron intake and hemoglobin in pregnancy with offspring vascular phenotypes and adiposity at age 10: findings from the Avon Longitudinal Study of Parents and Children. PLoS One.

[47] Paun A, Yau C, Danska JS (2017). The Influence of the Microbiome on Type 1 Diabetes. J Immunol.

[48] Gomez de Aguero M (2016). The maternal microbiota drives early postnatal innate immune development. Science.

[49] Hu Y (2015). Maternal Antibiotic Treatment Protects Offspring from Diabetes Development in Nonobese Diabetic Mice by Generation of Tolerogenic APCs. J Immunol.

[50] Needell JC (2017). Maternal treatment with short-chain fatty acids modulates the intestinal microbiota and immunity and ameliorates type 1 diabetes in the offspring. PLoS One.

[51] Dostal A (2012). Iron depletion and repletion with ferrous sulfate or electrolytic iron modifies the composition and metabolic activity of the gut microbiota in rats. J Nutr.

[52] Lee T (2017). Oral versus intravenous iron replacement therapy distinctly alters the gut microbiota and metabolome in patients with IBD. Gut.

[53] Berg, A. S., Inchley, C. S., Fjaerli, H. O., Leegaard, T. M. & Nakstad, B. Assessing Severity in Pediatric Pneumonia: Predictors of the Need for Major Medical Interventions. *Pediatr Emerg Care*, 10.1097/PEC.0000000000001179 (2017). 10.1097/PEC.0000000000001179 28538606

[54] Ronningen KS (2006). The biobank of the Norwegian Mother and Child Cohort Study: a resource for the next 100 years. Eur J Epidemiol.

[55] Skrivarhaug T, Stene LC, Drivvoll AK, Strøm H, Joner G (2014). Incidence of type 1 diabetes in Norway among children aged 0-14 years between 1989 and 2012: has the incidence stopped rising? Results from the Norwegian Childhood Diabetes Registry. Diabetologia.

[56] Irgens HU (2013). Prevalence of monogenic diabetes in the population-based Norwegian Childhood Diabetes Registry. Diabetologia.

[57] Meltzer HM, Brantsaeter AL, Ydersbond TA, Alexander J, Haugen M (2008). Methodological challenges when monitoring the diet of pregnant women in a large study: experiences from the Norwegian Mother and Child Cohort Study (MoBa). Matern Child Nutr.

[58] Brantsaeter AL (2007). Self-reported dietary supplement use is confirmed by biological markers in the Norwegian Mother and Child Cohort Study (MoBa). Ann Nutr Metab.

[59] Marild, K. *et al*. Midpregnancy and cord blood immunologic biomarkers, HLA genotype, and pediatric celiac disease. *J Allergy Clin Immunol*, 10.1016/j.jaci.2016.10.016 (2016). 10.1016/j.jaci.2016.10.016 27865861

[60] Mårild, K. *et al*. Midpregnancy and cord blood immunologic biomarkers, HLA genotype, and pediatric celiac disease. *J Allergy Clin Immunol*, 10.1016/j.jaci.2016.10.016 (2016). 10.1016/j.jaci.2016.10.016 27865861

[61] Dilthey A (2013). Multi-population classical HLA type imputation. PLoS Comput Biol.

[62] Aitken RJ, Mortimer GL, Gillespie KM (2016). Type 1 Diabetes High-Risk HLA Class II Determination by Polymerase Chain Reaction Sequence-Specific Primers. Methods Mol Biol.

[63] Alexander J, Kowdley KV (2009). HFE-associated hereditary hemochromatosis. Genet Med.

[64] Nielsen PB (2012). Sample-to-SNP kit: a reliable, easy and fast tool for the detection of HFE p.H63D and p.C282Y variations associated to hereditary hemochromatosis. Gene.

[65] Midttun Ø, Hustad S, Ueland PM (2009). Quantitative profiling of biomarkers related to B-vitamin status, tryptophan metabolism and inflammation in human plasma by liquid chromatography/tandem mass spectrometry. Rapid communications in mass spectrometry: RCM.

[66] Bibikova M (2011). High density DNA methylation array with single CpG site resolution. Genomics.

[67] Stanislawski MA (2017). Pre-pregnancy weight, gestational weight gain, and the gut microbiota of mothers and their infants. Microbiome.

[68] Dahl, C. *et al*. Preterm infants have distinct microbiomes, not explained by mode of delivery, less breastfeeding, or antibiotic exposure. *International journal of epidemiology*, In revision (2017). 10.1093/ije/dyy064 29688458

