# Supplementary material for: Prenatal iron exposure and childhood type 1 diabetes
Source: Sci Rep. 2018 Jun 13;8:9067. doi: 10.1038/s41598-018-27391-4 (PMC5998022; doi:10.1038/s41598-018-27391-4)
Supplement: Supplementary file 1 — Supplementary Information [file 41598_2018_27391_MOESM1_ESM.docx]

**Online supplement**

This appendix has been provided by the authors to give additional information about their work.

Supplement to: Ketil Størdal, Harry MacArdle, Helen Hayes, German Tapia, Marte Viken, Nicolai A. Lund-Blix, Margaretha Haugen, Geir Joner, Torild Skrivarhaug, Karl Mårild, Pål R. Njølstad, Merete Eggesbø, Siddhartha Mandal, Christian Page, Stephanie London, Benedicte Lie and Lars C. Stene.

**Prenatal iron exposure and childhood type 1 diabetes**

**Table of contents**

[Online methods 3](#_Toc510533544)

[Supplementary Table S1: Characteristics of participants by iron supplement use. 7](#_Toc510533545)

[Supplementary Table S2: Maternal anaemia, lowest haemoglobin, cumulative dose of iron supplements and risk of type 1 diabetes. 9](#_Toc510533546)

[Supplementary Table S3: Child HLA risk category, maternal use of iron supplements and risk of type 1 diabetes in the nested case-control sample. 10](#_Toc510533547)

[Supplementary Table S4: Childs use of iron supplements at age 6 and 18 months and risk of type 1 diabetes. 11](#_Toc510533548)

[Supplementary Table S5: Cord blood ferritin and soluble transferrin receptor by environmental determinants in controls*. 12](#_Toc510533549)

[Supplementary Table S6: Ferritin and soluble transferring receptor (mmol/L) in cord blood by *HFE* SNPs and genotypes for hemochromatosis in mother and child. 14](#_Toc510533550)

[Supplementary Table S7: Cord blood plasma ferritin and soluble transferring receptor by inflammatory markers in randomly selected controls. 15](#_Toc510533551)

[Supplementary Table S8: Bacterial diversity expressed as Shannon index by maternal iron supplement use in mothers and their infants born at term*. 16](#_Toc510533552)

[Supplementary Table S9: Mean difference in ratios of major microbial families in term babies by iron supplementation using linear regression models under a generalized estimating equation framework, separately for the first three time points and last three time points*. 17](#_Toc510533553)

[Supplementary Table S10: Analysis of short chain fatty acid concentrations during the first year of life in term infants by maternal use of iron supplements during pregnancy, using linear regressions adjusted for maternal BMI and maternal education*. 18](#_Toc510533554)

[Supplementary Figure S1: Use of iron supplements by week of pregnancy and lowest haemoglobin measured during week 0-30. 19](#_Toc510533555)

[Supplementary Figure S2. Association between SNPs proximal to the hemochromatosis associated *HFE* rs1800462 and cord blood DNA methylation at the five CpG sites most significantly associated with this SNP in the MoBa cohort. 20](#_Toc510533556)

[Supplementary Figure S3a-c: Post-hoc analysis of previously established non-HFE SNPs linked to iron status and genome wide cord blood DNA methylation in samples from the MoBa cohort. 23](#_Toc510533557)

[Supplementary Figure S4: Box-plot of cytokine z-scores (maternal samples approximately week 17 of pregnancy) by iron supplement use week 0-17 from randomly selected controls (n=519). 25](#_Toc510533558)

[Supplementary Figure S5: Concentrations of interferon (IFN)-γ and CCL4 in maternal mid-pregnancy samples, by maternal *HFE* (rs1800562) genotype. 26](#_Toc510533559)

[Supplementary Figure S6. Abundance of bacterial families (Operational Taxonomic Units, OTU) in fecal samples from a) mothers at delivery b) infant at age 10 days and c) infant at age 30 days, by maternal use of iron supplements (Iron: No/Yes). 27](#_Toc510533560)

[References 29](#_Toc510533561)

# Online methods

*Laboratory assays of iron in cord plasma*

Cord serum ferritin and serum soluble transferrin receptor (sTfR) concentrations were quantified at Rowett Institute, Aberdeen, UK using ELISA (DE4408; Demeditec Diagnostics, Germany and Human sTfR Quantikine IVD, DTFR1; R&D Systems, UK, respectively). Due to restrictions in sample volume, the sTfR assay was adjusted to 10µl of serum analysed whilst the ferritin kit was assayed according to manufacturer’s recommendations (20µl). All plates contained both intra- and inter-plate quality controls. sTfR levels were measured in nmol/L units and values converted to μg/L using a molecular weight of sTfR of 75,000 Da (R&D technical data sheet).

Ferritin was used as a measure of iron stores and sTfR was used as an indicator of iron deficiency; both are more sensitive indicators of iron status than dietary iron intake or haemoglobin concentration. Decreasing ferritin concentrations and increasing sTfR concentrations are evidence of declining status.

*Genotyping in the PAGE study*

All type 1 diabetes cases with available DNA from the mother (n=162) or the child (n=168) and a random sample from the whole cohort (n=511 pairs of mothers and children) were selected for genotyping in the PAGE study. We used a custom Illumina Golden Gate assay. SNPs (n=384) were selected based on prior knowledge on association to type 1 diabetes or celiac disease, or selected candidate gene SNPs for specific sub-studies as described elsewhere(*1*). We included in the Custom GoldenGate assay two coding variants in the *HFE* gene, rs1800562 (p.282Y) and rs1799945 (p.63D)(*2*), and the rs1799945 was additionally confirmed in a Taqman assay due to quality issues (as described below).

*Genotyping Quality control*

Both SNPs and samples underwent quality control (QC) before analysis. Some of the samples (n=45) were run as duplicates. The SNPs were clustered, assigned genotypes and exported from GenomeStudio, using a gene-call threshold of 0.25 for inclusion. Further QC was done in Stata, R and Plink. SNPs were excluded on the basis of deviation from Hardy-Weinberg Equilibrium in controls (n=27, with p≤0.01), ≥10 Mendelian errors (n=5), genotype success rate <80% (n=5) and aberrant clustering (visually inspected by two independent researchers, n=48). As we have mother-child dyads, Mendelian errors in this study can only be detected between homozygous mothers and children. Notably, many of the SNPs that failed due to improper clustering passed other software QC-tests. Overall, 65 SNPs were excluded (i.e. some SNPs failed in multiple tests) and constituted mostly HLA tag-SNPs excluded due to aberrant clustering (39 of 48) and deviation from Hardy-Weinberg Equilibrium (24 of 27).

Samples were excluded if they had GenCall score below 0.25, had ≥5% missing observations (after removing failed SNPs, n=70), failed to duplicate (≥10% discrepant calls, n=5) or had ≥10 Mendelian errors (n=13 mother/child dyads). The thresholds used in the QC was chosen *a priori* (GenCall threshold, Hardy-Weinberg Equilibrium, discrepancies), or *a posteriori* (Mendelian errors, genotype success rate) from plotting the data and observing which families gave rise to the Mendelian errors and missing calls in the dataset.

*Genotyping and methods for HLA prediction*

HLA genotype was imputed with the HLA*IMP:02 software (*3*) using 144 HLA tag SNPs, which was subsequently confirmed by classical HLA genotyping using allele specific PCR. Based on the previously established risk of T1D conferred by HLA genotype, we categorized genotypes into four groups: Protective (carrying at least one copy of HLA DQA1*01:02-DQB1*06:02-DRB1*15:01 [DQ6-DR15]), increased risk (at least one copy of HLA DQA1*03-DQB1*03:02-DRB1*04 [DQ8-DR4] or DQA1*05:01-DQB1*02:01 [DQ2-DR3], but not both haplotypes), high risk (heterozygous HLA DQ2-DR3/DQ8-DR4) or neutral (any other genotype).

Additionally, some of the case children had been genotyped at diagnosis at the Norwegian Childhood Diabetes Register. These data were used to verify our results, with good concordance between the HLA genotyping methods.

The two common variants in the *HFE* gene were identified by two tagSNPs as previously described (rs1800562 for p.282Y, rs1799945 for p.63D)(*4*). The two SNPs are in linkage equilibrium in the CEU population (r^2^=0.008) (*5*). The rs1799945 failed QC due to overlapping clusters. Retyping of this SNP was performed by allelic discrimination on an ABI7300 using a custom TaqMan® SNP genotyping assay. Interestingly, the results were largely similar, with 96.7% having the same genotype, 3.3% having one allele misclassified and none being grossly misclassified with both alleles different.

The *HFE* variants could be present in the mother, the child or both, and we studied the association with the *HFE* gene variants separately and with reciprocal adjustment for maternal and fetal genotype.

A formal test for linkage disequilibrium in a reference population (CEU) between the main HLA risk allele and *HFE* genotypes because of the proximity in location on chromosome 6p demonstrated linkage equilibrium between rs1800562 and rs1799945 and the HLA tag SNP rs2187668 (r^2^=0.0066 and 0.0006, respectively) (*5*).

*Analyses of mid-pregnancy maternal samples for cytokines*

We selected a limited set of cytokines based on prior association to either celiac disease or T1D, and to ensure representation of Th1, Th2, Th17, and Treg immune responses in the adaptive immune system. We avoided cytokines known or suspected to be present in undetectable concentrations in most healthy individuals, or to give unreliable measurements e.g. due to pre-analytical factors. In total we measured 18 cytokines in maternal mid-pregnancy plasma using Bio-Plex protein array systems (Bio-Rad, Hercules, CA), based on xMAP technology (Luminex, Austin, TX) as previously described (*1*). The cytokines included are shown in Supplementary Figure 3.

Concentrations were calculated from a standard curve generated from standards provided by the assay manufacturer. The lower limit of quantification ranged from 0.02 (IL-4) to 1.35 (CCL2) pg/mL, and the inter-plate mean coefficient of variation (CV) across all dilutions ranged from 2.2% (CCL4) to 8.0% (IL-4). Further details of limits of quantification, inter- and intra-assay CVs are given in (*1*).

Umbilical-cord plasma concentrations of neopterin, tryptophan and kynurenine were measured using a high-throughput liquid chromatography tandem mass-spectrometry at the Bevital laboratory (Bergen, Norway) (*6*), which have previously demonstrated acceptable pre-analytical stability (*7*).

*Analyses of cord samples for DNA methylation*

We analysed 1,062 participants who had available cord blood samples and non-missing data covariates, as described previously (*8*). Bisulfite conversion was performed using the EZ-96 DNA Methylation kit (Zymo Research Corporation, Irvine, CA) according to manu­facturer instructions. We measured methylation at 485,577 CpGs in cord blood using the Illumina’s Infinium HumanMethylation450 BeadChip (*9*).

*Analyses of fecal samples for bacterial strains*

Fecal samples were collected at 4 days after birth from both mother and child and thereafter only from the child at day 10, 1 and 4 months, and 1 and 2 years as part of the NoMIC cohort (*10*). In total, 2752 fecal samples were obtained. Seventy-two percent of the children had Illumina data on five or more time points and 37 percent had data on all age points. Altogether 1691 samples were available from the 367 term babies included in this analysis. DNA was extracted from the samples, and subjected to barcoded sequencing of the V4 region of the 16S rRNA gene using an Illumina HiSeq 2000 machine (*10*).

*Analyses of fecal samples for short-chain fatty acids*

Two hundred and fifty nine term infants had samples available for chemical analyses of eight short chain fatty acids (SCFA). Samples were analysed in two different laboratories, using gas chromatography, a capillary column and quantified using internal standardization and flame ionization detection (*11*). SCFA levels with undetectable levels were replaced by a random number below lowest observed value, using the Stata command “runiform”.

*Statistical analysis of questionnaire data, biomarkers of iron and genotype:*

We used Cox proportional hazard regression analysis to examine the association of maternal iron supplement use with diagnosis of type 1 diabetes, reporting hazard ratios (HRs) with 95% confidence intervals. Follow-up time was counted from date of birth to the first date of type 1 diabetes diagnosis, death, emigration, or to end of follow-up (May 1st, 2017) whatever came first. We used robust cluster variance estimation to correct for within-family correlation. The proportional hazard assumption of the Cox model was assessed by visual inspection of plots and tested using Schoenfeld residuals, which indicated no deviations from this model assumption.

We defined statistical significance as 95% confidence intervals for the hazard ratio not including 1.00.

We used logistic regression of the nested case-control data to study the association of biomarkers of iron stores and *HFE* variants with type 1 diabetes.

For missing covariates we used chained multiple imputation with 25 imputed sets. In a sensitivity analysis we performed the main analysis in complete cases. Additionally, we restricted the analysis to pregnancies without anaemia and to families with Norwegian ethnicity. Finally, we stratified the analyses by four categories of HLA risk to assess potential heterogeneity of associations.

We used Stata (V14, StataCorp., College Station, TX, US) for the statistical analyses.

*Statistical analysis of cytokines:*

The number of markers was restricted to 20 (18 in maternal samples used in this study) to limit multiple testing problems. Z-scores for each cytokine were calculated and used in the analysis of individual cytokines and a sum z-score for immunological pathways. We tested the association with iron supplement use for each cytokine separately, aiming primarily to test the hypothesis whether iron supplement use was associated with macrophage polarization of the M1 pathway. We thus grouped the subsets of cytokines belonging to the M1 pathway and added individual z-scores to a common pathway z-score.

We decided to study whether iron supplement use predicted cytokine concentrations on randomly selected controls, and thereafter in the whole cohort adjusted for case status as the M1 pathway cytokines have been found to be associated with type 1 diabetes. In an exploratory analysis, we tested the effect of maternal SNPs rs1800562 and rs1799945 on cytokine levels.

*Statistical analysis of DNA methylation:*

Illumina’s GenomeStudio® Methylation module version 1.0 (Illumina Inc.) was used to calculate the methylation level at each CpG as the beta-value [β = intensity of the methylated allele (M) / (intensity of the unmethylated allele (U) + intensity of the methylated allele (M) + 100)] . Beta-values were then transformed to obtain the log ratio, defined as log[β/(1 – β)].

*Statistical analysis of microbiome:*

Diversity in gut microbial communities were measured using Shannon's diversity index at all time points. For maternal samples (one per mother), diversity was studied using a standard linear regression. Difference in diversity profiles over time were studied using longitudinal linear models with an autoregressive correlation structure for intra-individual correlations within infants.

Microbial composition was studied on the basis of relative abundance at the family level for each time point. Due to the compositionality of relative abundance of microbes (sum of relative abundances being equal to unity), standard statistical methods are not applicable on the relative abundance of particular microbes. Hence, we studied the mean difference in ratios of major microbial families for those exposed to iron supplementation and not, using linear regression models. In addition, differentially abundant microbial OTUs were also identified in maternal and infant samples using ANCOM which accounts for compositional structure in microbial communities and is free from distributional assumptions.

Differences in concentrations of 8 short chain fatty acids between levels of iron supplementation were studied at five early life time points (4 days, 10 days, 1 month and 4 months) using linear regressions adjusted for mode of delivery, gestational age and months of any breastfeeding.

|  | Iron supplement used in pregnancy % (n) | | | |  |
| --- | --- | --- | --- | --- | --- |
|  | **No use** | **<17 weeks GA only** | **>17 weeks GA only** | **Both </> 17 weeks GA** | p-value |
| Whole cohort | 36 (33,918) | 4 (3,368) | 27 (25,897) | 33 (31,026) |  |
| Year of childbirth | | | | | <0.001 |
| 2000-2005 | 33 (16,470) | 3 (1,587) | 29 (14,818) | 35 (17,424) |  |
| 2006-2009 | 40 (17,448) | 4 (1,781) | 25 (11,079) | 31 (13,602) |  |
| Smoking * | | | | | <0.001 |
| No | 35 (29,948) | 3 (2,917) | 27 (23,182) | 34 (28,408) |  |
| Occasionally | 38 (637) | 4 (75) | 27 (460) | 31 (522) |  |
| Yes | 42 (2,884) | 4 (289) | 28 (1,897) | 26 (1,779) |  |
| Parity | | | | | <0.001 |
| 0 | 34 (14,692) | 4 (1,732) | 27 (11,364) | 35 (14,999) |  |
| 1 | 37 (12,173) | 3 (1,084) | 28 (9,487) | 32 (10,581) |  |
| ≥ 2 | 39 (7,053) | 3 (552) | 28 (5,046) | 30 (5,446) |  |
| Maternal age | | | | | <0.001 |
| < 25 | 37 (3,718) | 5 (491) | 28 (2,780) | 30 (3,025) |  |
| 25-34 | 36 (24,443) | 4 (2,431) | 27 (18,494) | 33 (22,358) |  |
| ≥ 35 | 35 (5,757) | 3 (446) | 28 (4,623) | 34 (5,643) |  |
| Maternal education^†^ | | | | | <0.001 |
| Lowest | 38 (13,198) | 4 (1,389) | 28 (9,684) | 30 (10,242) |  |
| Intermediate | 35 (13,343) | 3 (1,286) | 27 (10,441) | 34 (13,085) |  |
| Highest | 34 (7,200) | 3 (677) | 27 (5,647) | 36 (7,585) |  |
| Pre-pregnancy body mass index^‡^ | | | | | <0.001 |
| < 20 | 31 (3,505) | 3 (390) | 29 (3,331) | 37 (4,258) |  |
| 20-25 | 35 (17,912) | 4 (1,826) | 28 (14,262) | 34 (17,468) |  |
| 25-29.99 | 39 (7,796) | 4 (738) | 27 (5,416) | 31 (6,148) |  |
| ≥ 30 | 43 (3,745) | 4 (331) | 25 (2,145) | 29 (2,523) |  |
| Haemoglobin – lowest measured^§^ | |  |  |  | <0.001 |
| < 10.5 | 20 (2,089) | 3 (282) | 39 (4,125) | 39 (4,109) |  |
| 10.5-11.4 | 32 (7,693) | 3 (778) | 30 (7,207) | 35 (8,545) |  |
| 11.5-12.4 | 38 (9,873) | 4 (962) | 25 (6,556) | 33 (8,697) |  |
| ≥ 12.5 | 43 (4,964) | 4 (445) | 23 (2,657) | 30 (3,533) |  |
| Diagnosed maternal anaemia | |  |  |  | <0.001 |
| No | 37 (33,389) | 4 (3,140) | 28 (25,170) | 31 (27,912) |  |
| Yes | 12 (529) | 5 (228) | 16 (727) | 68 (3,114) |  |
| Maternal diabetes | | | | | 0.04 |
| No | 36 (33,724) | 4 (3,350) | 28 (25,790) | 33 (30,851) |  |
| Yes | 39 (194) | 4 (18) | 22 (107) | 35 (175) |  |
| Maternal celiac disease | | | | | <0.001 |
| No | 36 (33,716) | 4 (3,343) | 28 (25,713) | 33 (30,686) |  |
| Yes | 27 (202) | 3 (25) | 25 (184) | 45 (340) |  |
| Maternal *HFE* haplotype^\|\|^  (controls from the genotyped nested case control study, n=513) | | | | | 0.11 |
| Standard | 41 (150) | 4 (16) | 23 (83) | 32 (118) |  |
| Intermediate | 32 (45) | 1 (2) | 30 (42) | 36 (50) |  |
| High | 1. (14) | 1 (14) | 2 (29) | 3 (43) |  |
| Birth weight |  |  |  |  | 0.007 |
| <2500 | 36 (1,265) | 3 (122) | 26 (924) | 34 (1,191) |  |
| 2500-3499 | 37 (13,177) | 4 (1,300) | 27 (9,655) | 33 (11,738) |  |
| 3500-4499 | 36 (17,992) | 4 (1,811) | 28 (14,169) | 33 (16,721) |  |
| >4500 | 36 (1,463) | 4 (3,366) | 28 (1,135) | 33 (1,371) |  |
| Gestational age, weeks | |  |  |  | 0.087 |
| < 37 | 36 (31,786) | 4 (3,167) | 28 (24,373) | 33 (29,058) |  |
| ≥ 37 | 36 (1,976) | 3 (185) | 26 (1,418) | 34 (1,845) |  |

# Supplementary Table S1: Characteristics of participants by iron supplement use.

* Smoking missing for 1,211

^†^ Maternal education missing for 432

^‡^ Pre-pregnancy BMI missing for 2,415

^§^ Haemoglobin missing for 21,694

^||^ Standard: Wild type *HFE* allele at both loci (C282 and H63).

Intermediate: p.63D/63D homozygotes and p.63D or p.282Y heterozygotes

High: p.282Y homozygous or p.282Y/p.63D compound heterozygotes.

# Supplementary Table S2: Maternal anaemia, lowest haemoglobin, cumulative dose of iron supplements and risk of type 1 diabetes.

|  | **Type 1 diabetes** | | **Unadjusted hazard ratio (95% CI)** | **Adjusted hazard ratio**  **(95% CI) ^*^** |
| --- | --- | --- | --- | --- |
|  | **No**  **n= 93,836 (%)** | **Yes**  **n=373 (%)** |  |  |
| Additional analyses by cause of iron supplement use | | | | |
| Maternal anaemia treated during pregnancy | 1,866 (2.0) | 5 (1.3) | 0.63 (0.26-1.53) | 0.68 (0.28-1.63) |
| Haemoglobin <10.5^‡^ | 10,561 (14.6) | 44 (16.9) | 1.07 (0.79-1.46) | 1.13 (0.83-1.54) |
| Cumulative dose of iron supplements during pregnancy^§^ | | | | |
| No use | 33 806 (36.0) | 112 (30.0) | 1 (ref.) | 1 (ref.) |
| Low dose | 25,556 (27.2) | 112 (30.0) | 1.31 (1.01-1.70) | 1.37 (1.05-1.78) |
| Intermediate | 18,316 (19.5) | 84 (22.5) | 1.25 (0.94-1.66) | 1.30 (0.98-1.73) |
| High dose | 16,158 (17.2) | 65 (17.4) | 1.23 (0.90-1.67) | 1.31 (0.96-1.79) |
| Estimated daily intake of iron from foods week 22 of pregnancy^\|\|^ | | | | |
| Iron from food (mg/d, SD) | 11.2 (3.8) | 11.1 (3.5) |  |  |
| Per mg/d change |  |  | 1.00 (0.97, 1.02) | 1.00 (0.97, 1.03) |
| 0-25 centile | 20,882 (24.9) | 84 (25.9) | 1 (ref.) | 1 (ref.) |
| 25-50 centile | 20,963 (25.0) | 79 (24.4) | 0.94 (0.69-1.28) | 0.96 (0.70-1.30) |
| 50-75 centile | 20,946 (25.0) | 77 (23.8) | 0.92 (0.68-1.26) | 0.95 (0.70-1.30) |
| >75 centile | 20,983 (25.1) | 84 (25.9) | 1.01 (0.75-1.37) | 1.05 (0.77-1.42) |

**^*^** Adjusted for maternal age and education, parity, smoking, birth weight, prematurity, pre-pregnancy BMI, mode of delivery, diagnosed maternal anaemia, maternal type 1 diabetes and celiac disease.

**^†^** p-values for category vs reference category. Shaded areas show p-value for trend.

**^‡^** Missing haemoglobin for 82 cases and 21,623 non-cases.

^§^ Low dose: 0.1-2.1 g; Intermediate dose: 2.1-3.6 g; High dose: >3.6 g, the cut-off from 50^th^ and 75^th^ centile among users of iron-containing supplements.

^||^ n=84,098. 0-25 centile: 0.001-8.9 mg/d; 25-50 centile: 8.9-10.9 mg/d; 50-75 centile: 10.9-13.3 mg/d; >75 centile: >13.3 mg/d.

# Supplementary Table S3: Child HLA risk category, maternal use of iron supplements and risk of type 1 diabetes in the nested case-control sample.

| Child HLA type | Did not develop type 1 diabetes | Developed type 1 diabetes | Odds ratio  (95% CI) | Used iron | Did not develop type 1 diabetes | Developed type 1 diabetes | Odds ratio (95% CI) for use of iron supplements^\|\|^ |
| --- | --- | --- | --- | --- | --- | --- | --- |
|  | n=493 (%) | n=168 (%) |  |  | n=488 (%) | n=153 (%) |  |
| High-risk^*^ | 31 (6) | 69 (41) | 46.3  (17.2, 125) | Yes | 16 (52) | 41 (59) | 1.37 (0.59, 3.2) |
|  |  |  |  | No | 15 (48) | 28 (41) |  |
| Intermediate^†^ | 200 (41) | 91 (54) | 9.5  (3.7, 24.0) | Yes | 126 (63) | 61 (67) | 1.19 (0.71, 2.0) |
|  |  |  |  | No | 74 (37) | 30 (33) |  |
| Neutral^‡^ | 104 (21) | 5 (3) | 1 (ref.) | Yes | 63 (61) | 4 (80) | 2.6 (0.28, 24.1) |
|  |  |  |  | No | 41 (39) | 1 (20) |  |
| Protective^§^ | 158 (32) | 3 (2) | 0.4  (0.1, 1.7) | Yes | 98 (62) | 2 (67) | 1.22 (0.11, 13.8) |
|  |  |  |  | No | 60 (38) | 1 (33) |  |

^*^ heterozygous HLA DQ2.5-DR3/DQ8-DR4

^†^ at least one copy of HLA DQA1*03-DQB1*03:02 [DQ8-DR4] or DQA1*05:01-DQB1*02:01 [DQ2.5-DR3], but not both haplotypes

^‡^ any other genotype

^§^ carrying at least one copy of HLA DQA*01:02-DQB*06:02-DRB*15:01 [DQ6-DR15]

**^||^** Test for heterogeneity of odds ratios across HLA genotype category (interaction): p=0.97.

# Supplementary Table S4: Childs use of iron supplements at age 6 and 18 months and risk of type 1 diabetes.

| Childs use of iron supplement  < age 6 months | Type 1 diabetes | | Unadjusted hazard ratio (95% CI) | Adjusted hazard ratio (95% CI)* |
| --- | --- | --- | --- | --- |
|  | **No**  **n= 84,757 (%)** | **Yes**  **n=304 (%)** |  |  |
| Yes | 3,603 (4.3) | 16 (4.8) | 1.22 (0.75-2.00) | 1.43 (0.80-2.56) |
| No | 81,121 (95.8) | 321 (95.3) | 1 (ref.) | 1 (ref.) |
| Childs use of iron supplement  < age 18 months | **Type 1 diabetes** | |  |  |
|  | **No**  **n=72,907 (%)** | **Yes**  **n=265 (%)** |  |  |
| Yes | 986 (1.4) | 5 (1.7) | 1.19 (0.49-2.88) | 1.20 (0.49-2.91) |
| No | 71,894 (98.7) | 287 (98.3) | 1 (ref.) | 1 (ref.) |
| Any use by the child from 0-18 months | **Type 1 diabetes** | |  |  |
|  | **No**  **n=71,252 (%)** | **Yes**  **n=261 (%)** |  |  |
| Yes | 4,392 (6.2) | 20 (6.9) | 1.22 (0.78-1.90) | 1.36 (0.82-2.25) |
| No | 66,833 (93.8) | 268 (93.1) | 1 (ref.) | 1 (ref.) |

* Adjusted for maternal age and education, parity, smoking, birth weight, prematurity, pre-pregnancy BMI, mode of delivery, diagnosed maternal anaemia, maternal type 1 diabetes and celiac disease.

|  | n | Mean cord blood ferritin (95% CI) nmol/l * | Change per category  (95% CI) nmol/l | R^2 †^ | Mean cord blood sTfR (95% CI) nmol/l * | Change per category  (95% CI) nmol/l | R^2 †^ |
| --- | --- | --- | --- | --- | --- | --- | --- |
| *Any use of maternal iron supplement in pregnancy* | | | |  |  |  |  |
| No | 194 | 166 (146, 186) | 2.9 (-18, 24) | 0.0001 | 2.9 (2.7, 3.0) | -0.12 (-0.29, 0.05) | 0.004 |
| Yes | 306 | 169 (157, 180) |  |  | 2.8 (2.7, 2.9) |  |  |
| *Maternal iron intake from foods* | | |  |  |  |  |  |
| Lowest quartile | 120 | 163 (145, 181) | -4 (-14, 5) | 0.002 | 2.9 (2.7, 3.1) | -0.1 (-0.2, 0.0) | 0.01 |
| 2^nd^ quartile | 114 | 188 (161, 215) |  |  | 2.8 (2.6, 3.0) |  |  |
| 3^rd^ quartile | 118 | 168 (148, 189) |  |  | 2.8 (2.6, 3.0) |  |  |
| Upper quartile | 101 | 154 (137, 171) |  |  | 2.6 (2.5, 2.8) |  |  |
| *Maternal anemia ≤ 17 weeks of pregnancy* | | |  |  |  |  |  |
| No | 445 | 168 (158, 179) | -28 (-74, 17) | 0.003 | 2.8 (2.7, 2.9) | -0.2 (-0.6, 0.1) | 0.003 |
| Yes | 24 | 140 (108, 171) |  |  | 2.6 (2.2, 2.9) |  |  |
| *Maternal pre-pregnant BMI* | | |  |  |  |  |  |
| <20 | 54 | 178 (150, 205) | -13 (-26, 1) | 0.0072 | 2.4 (2.2, 2.6) | 0.3 (0.2, 0.4) | 0.06 |
| 20-25 | 278 | 170 (156, 185) |  |  | 2.8 (2.7, 3.1) |  |  |
| 25-30 | 92 | 164 (144, 185) |  |  | 2.9 (2.7, 3.1) |  |  |
| >30 | 33 | 131 (104, 158) |  |  | 3.5 (3.0, 4.0) |  |  |
| *Maternal age* | |  |  |  |  |  |  |
| <25 | 61 | 185 (146, 224) | -14 (-33, 6) | 0.0037 | 2.9 (2.7, 3.1) | 0.0 (-0.2, 0.1) | 0.0004 |
| 25-34 | 360 | 167 (155, 179) |  |  | 2.8 (2.7, 2.9) |  |  |
| ≥35 | 79 | 157 (132, 182) |  |  | 2.8 (2.6, 3.0) |  |  |
| *Maternal parity* | |  |  |  |  |  |  |
| 0 | 224 | 178 (160, 195) | -3 (-16, 11) | 0.0003 | 2.8 (2.7, 3.0) | 0.0 (-0.1, 0.1) | 0.0001 |
| 1 | 183 | 147 (134, 160) |  |  | 2.7 (2.6, 2.9) |  |  |
| ≥2 | 93 | 184 (158, 210) |  |  | 2.8 (2.6, 3.0) |  |  |
| *Period of birth* | |  |  |  |  |  |  |
| 1999-2005 | 286 | 158 (145, 170) | 23 (2, 44) | 0.009 | 2.8 (2.7, 2.9) | 0.1 (-0.1, 0.2) | 0.0017 |
| 2006-2009 | 214 | 181 (163, 198) |  |  | 2.8 (2.7, 3.0) |  |  |
| *Maternal smoking* | |  |  |  |  |  |  |
| None | 411 | 168 (158, 179) | -5 (-23, 12) | 0.0007 | 2.8 (2.7, 2.8) | 0.2 (0.1, 0.4) | 0.015 |
| Occasionally | 15 | 160 (112, 208) |  |  | 3.3 (2.6, 3.9) |  |  |
| Daily | 40 | 159 (115, 203) |  |  | 3.1 (2.8, 3.4) |  |  |

# Supplementary Table S5: Cord blood ferritin and soluble transferrin receptor by environmental determinants in controls*.

* Ferritin and sTfR concentrations in cord plasma were, as expected, negatively correlated (Spearman’s rho -0.20).

^†^ Proportion of variance explained by the predictor variable.

# Supplementary Table S6: Ferritin and soluble transferring receptor (mmol/L) in cord blood by *HFE* SNPs and genotypes for hemochromatosis in mother and child.

| SNPs | Geno-type | | n | Mean cord blood ferritin (95% CI) | Change per allele  (95% CI) | R^2^ * | Mean cord blood sTfR (95% CI) | Change per allele  (95% CI) | R^2^ * |
| --- | --- | --- | --- | --- | --- | --- | --- | --- | --- |
| *Per individual allele* | | | |  |  |  |  |  |  |
| *Maternal* |  | |  |  |  |  |  |  |  |
| *rs1800562* | GG | | 430 | 170 (158, 181) | -2 (-35, 31) | 0.0000 | 2.8 (2.7, 2.9) | 0.1 (-0.1, 0.4) | 0.0017 |
| (P.282Y) | AG | | 51 | 159 (128, 190) |  |  | 2.9 (2.7, 3.2) |  |  |
|  | AA | | 1 | 381 |  |  | 2.5 |  |  |
| *rs1799945* | CC | | 394 | 165 (154, 176) | 15 (-11, 42) | 0.0028 | 2.8 (2.7, 2.9) | 0.0 (-0.2, 0.2) | 0.0005 |
| (P.63D) | CG | | 83 | 184 (155, 213) |  |  | 2.8 (2.6, 3.0) |  |  |
|  | GG | | 3 | 150 (18, 282) |  |  | 3.3 (1.1, 5.4) |  |  |
| *Child* |  | |  |  |  |  |  |  |  |
| *rs1800562* | GG | | 427 | 168 (157, 180) | 6 (-25, 37) | 0.0003 | 2.8 (2.7, 2.9) | -0.1 (-0.3, 0.2) | 0.0011 |
| (P.282Y) | AG | | 51 | 164 (137, 191) |  |  | 2.7 (2.5, 3.0) |  |  |
|  | AA | | 3 | 265 (0, 745) |  |  | 2.6 (0.4, 4.8) |  |  |
| *rs1799945* | CC | | 369 | 170 (158, 183) | -3 (-26, 20) | 0.0002 | 2.8 (2.7, 2.9) | 0.1 (-0.1, 0.2) | 0.0007 |
| (P.63D) | CG | | 105 | 164 (146, 183) |  |  | 2.8 (2.7, 3.0) |  |  |
|  | GG | | 7 | 176 (88, 265) |  |  | 2.8 (2.1, 3.6) |  |  |
| *Genotype risk category for hemochromatosis* | | | | |  |  |  |  |  |
| *Maternal* | |  |  |  |  |  |  |  |  |
| Low ^†^ | |  | 347 | 166 (154, 178) | 11 (-11, 33) | 0.0020 | 2.8 (2.7, 2.9) | 0.1 (-0.1, 0.2) | 0.0016 |
| Intermediate ^‡^ | |  | 127 | 170 (148, 191) |  |  | 2.8 (2.7, 3.0) |  |  |
| High ^§^ | |  | 6 | 252 (102, 402) |  |  | 3.1 (2.3, 3.9) |  |  |
| *Child* | |  |  |  |  |  |  |  |  |
| Low ^†^ | |  | 323 | 170 (155, 184) | -1 (-21, 20) | 0.0000 | 2.8 (2.7, 2.9) | 0.0 (-0.2, 0.2) | 0.0000 |
| Intermediate ^‡^ | |  | 147 | 164 (149, 180) |  |  | 2.8 (2.7, 3.0) |  |  |
| High ^§^ | |  | 11 | 193 (103, 283) |  |  | 2.5 (2.0, 3.0) |  |  |

* Proportion of variance explained by the predictor variable.

^†^ Low: Homozygous GG at rs1800562 and CC at rs1799945

**^‡^** Intermediate: Homozygous for the p.63D variant, or heterozygous at either p.282Y or p.63D.

^§^ High: Homozygous for the p.282Y variant, or compound heterozygous for the p.282Y and p.63D variants.

# Supplementary Table S7: Cord blood plasma ferritin and soluble transferring receptor by inflammatory markers in randomly selected controls.

|  | |  | Ferritin | |  | |  | |  | | sTfR | | |  | |  |
| --- | --- | --- | --- | --- | --- | --- | --- | --- | --- | --- | --- | --- | --- | --- | --- | --- |
|  | |  |  | |  | |  | |  | |  | | |  | |  |
|  | n | Change per unit increase  (95% CI) nmol/l | |  | | R^2 †^ | | n | | Change per unit increase  (95% CI) mg/ml | |  | | | | R^2 †^ |
| Kynurenine/tryptophan ratio  (mean 48.9 nmol/μmol,SD 13.3) | | | |  | |  | |  | |  | | | | |  |  |
|  | 501 | 0.99 (0.30, 1.69) | |  | | 0.0155 | | 511 | | 0.005 (-0.00, 0.01) | |  | 0.0067 | | | |
| Neopterin  (mean 29.2 nmol/l, SD 8.8) | |  | |  | |  | |  | |  | |  | | | |  |
|  | 496 | 1.00 (-0.21, 2.21) | |  | | 0.0053 | | 511 | | 0.005 (-0.00, 0.01) | |  | | | | 0.040 |

# Supplementary Table S8: Bacterial diversity expressed as Shannon index by maternal iron supplement use in mothers and their infants born at term*.

|  | Regression coefficient | Standard error | p-value |
| --- | --- | --- | --- |
| Mother; at delivery |  |  |  |
| Intercept | 3.76 | 0.060 |  |
| Maternal iron supplement | 0.15 | 0.081 | 0.067 |
| Baby – change from 30 to 120 days | |  |  |
| Intercept | 0.119 | 0.048 |  |
| Maternal iron supplement | -0.014 | 0.065 | 0.83 |
| Baby – day 4 |  |  |  |
| Intercept | 2.01 | 0.038 |  |
| Maternal iron supplement | 0.004 | 0.052 | 0.94 |
| Baby – day 10 |  |  |  |
| Intercept | 2.02 | 0.035 |  |
| Maternal iron supplement | 0.033 | 0.048 | 0.50 |
| Baby – day 30 |  |  |  |
| Intercept | 2.04 | 0.039 |  |
| Maternal iron supplement | -0.026 | 0.054 | 0.63 |
| Baby – day 120 |  |  |  |
| Intercept | 2.18 | 0.040 |  |
| Maternal iron supplement | -0.032 | 0.055 | 0.56 |

*Number of term babies is 305, 298, 310 and 288 at 4 days, 10 days, 1 month and 4 months.

# Supplementary Table S9: Mean difference in ratios of major microbial families in term babies by iron supplementation using linear regression models under a generalized estimating equation framework, separately for the first three time points and last three time points*.

| **Overall first month^†^** |  |  |  |
| --- | --- | --- | --- |
| Lactobacillaceae:Enterobacteriaceae | Estimate | 95% CI | p-value |
| Intercept | -8.30 |  |  |
| Iron supplement | 0.93 | -0.45, 2.30 | 0.19 |
| Time | 0.077 | 0.039, 0.12 | <0.001 |
| Iron supplement * time | -0.007 | -0.068, 0.054 | 0.82 |
| Bifidobacteriaceae:Enterobacteriaceae |  |  |  |
| Intercept | -0.46 |  |  |
| Iron supplement | 0.60 | -0.48, 1.68 | 0.27 |
| Time | 0.040 | 0.015, 0.065 | 0.002 |
| Iron supplement * time | -0.005 | -0.045, 0.036 | 0.82 |
| Bacteroidaceae:Clostridiaceae |  |  |  |
| Intercept | 1.50 |  |  |
| Iron supplement | -0.72 | -2.64, 1.21 | 0.47 |
| Time | -0.085 | -0.14, -0.029 | 0.003 |
| Iron supplement * time | 0.11 | 0.035, 0.19 | 0.004 |
| Bacteroidaceae:Enterobacteriaceae |  |  |  |
| Intercept | -5.72 |  |  |
| Iron supplement | 1.02 | -0.47, 2.50 | 0.18 |
| Time | -0.016 | -0.054, 0.022 | 0.41 |
| Iron supplement * time | 0.022 | -0.032, 0.075 | 0.43 |
| **Overall 4 months – 2 years**^‡^ |  |  |  |
| Lactobacillaceae:Enterobacteriaceae |  |  |  |
| Intercept | -4.98 |  |  |
| Iron supplement | 0.17 | -1.33, 1.67 | 0.83 |
| Time | 0.003 | 0.000, 0.006 | 0.03 |
| Iron supplement * time | -0.002 | -0.006, 0.002 | 0.34 |
| Bifidobacteriaceae:Enterobacteriaceae |  |  |  |
| Intercept | 0.86 |  |  |
| Iron supplement | 0.11 | -0.77, 0.99 | 0.80 |
| Time | 0.006 | 0.004, 0.008 | <0.001 |
| Iron supplement * time | -0.001 | -0.004, 0.001 | 0.28 |
| Bacteroidaceae:Clostridiaceae |  |  |  |
| Intercept | -2.98 |  |  |
| Iron supplement | 2.47 | 0.65, 4.29 | 0.008 |
| Time | 0.002 | -0.001, 0.004 | 0.19 |
| Iron supplement * time | -0.003 | -0.007, 0.000 | 0.066 |
| Bacteroidaceae:Enterobacteriaceae |  |  |  |
| Intercept | -6.61 |  |  |
| Iron supplement | 1.08 | -0.44, 2.61 | 0.16 |
| Time | 0.014 | 0.011, 0.017 | <0.001 |
| Iron supplement * time | -0.002 | -0.005, 0.001 | 0.26 |

***** Number of term babies is 305, 298, 310, 288, 223 and 99 at 4 days, 10 days, 1 month, 4 months, 1 year and 2 years.

**^†^** Stools were collected at day 4, 10 and 30

^‡^ Stools were collected at age 4, 12 and 24 months

# Supplementary Table S10: Analysis of short chain fatty acid concentrations during the first year of life in term infants by maternal use of iron supplements during pregnancy, using linear regressions adjusted for maternal BMI and maternal education*.

| Timepoint/type of SCFA | Regression coefficient | 95% CI | p-value |
| --- | --- | --- | --- |
| Day 4 |  |  |  |
| 1. acetic | 1.74 | -48.8, 52.3 | 0.94 |
| 2. propionic | -1.59 | -7.15, 3.98 | 0.56 |
| 3. butyric | -0.36 | -1.63, 0.91 | 0.56 |
| 4. isobutyric | 0.045 | -0.15, 0.24 | 0.64 |
| 5. valeric | 0.008 | -0.074, 0.091 | 0.84 |
| 6. isovaleric | 0.026 | -0.07, 0.12 | 0.58 |
| 7. caproic | 0.15 | 0.022, 0.28 | 0.023 |
| 8. isocaproic | -0.039 | -0.14, 0.062 | 0.43 |
| Day 10 |  |  |  |
| 1. acetic | -3.67 | -38.2, 30.8 | 0.83 |
| 2. propionic | 0.037 | -4.64, 4.71 | 0.99 |
| 3. butyric | -0.40 | -2.05, 1.25 | 0.63 |
| 4. isobutyric | 0.008 | -0.35, 0.37 | 0.97 |
| 5. valeric | 0.015 | -0.046, 0.077 | 0.62 |
| 6. isovaleric | 0.07 | -0.49, 0.63 | 0.81 |
| 7. caproic | 0.00 | -0.018, 0.017 | 0.97 |
| 8. isocaproic | -0.002 | -0.087, 0.084 | 0.97 |
| Day 30 |  |  |  |
| 1. acetic | 5.85 | -19.8, 31.5 | 0.65 |
| 2. propionic | -1.91 | -7.98, 4.16 | 0.53 |
| 3. butyric | 0.46 | -1.01, 1.92 | 0.54 |
| 4. isobutyric | -0.001 | -0.21, 0.21 | 0.99 |
| 5. valeric | 0.11 | -0.078, 0.30 | 0.25 |
| 6. isovaleric | -0.065 | -0.31, 0.18 | 0.60 |
| 7. caproic | 0.008 | -0.009, 0.024 | 0.36 |
| 8. isocaproic | 0.01 | -0.021, 0.041 | 0.51 |
| Day 120 |  |  |  |
| 1. acetic | -5.44 | -30.6, 19.7 | 0.67 |
| 2. propionic | -0.67 | -6.50, 5.16 | 0.82 |
| 3. butyric | -1.43 | -3.45, 0.58 | 0.16 |
| 4. isobutyric | -0.15 | -0.59, 0.29 | 0.49 |
| 5. valeric | -0.038 | -0.17. 0.095 | 0.57 |
| 6. isovaleric | 0.035 | -0.67, 0.74 | 0.92 |
| 7. caproic | 0.009 | -0.025, 0.043 | 0.59 |
| 8. isocaproic | 0.007 | -0.088, 0.10 | 0.89 |
| Day 365 |  |  |  |
| 1. acetic | 0.076 | -12.4, 12.6 | 0.99 |
| 2. propionic | 1.16 | -2.30, 4.63 | 0.51 |
| 3. butyric | 0.75 | -2.85, 4.35 | 0.68 |
| 4. isobutyric | -0.18 | -0.56, 0.20 | 0.34 |
| 5. valeric | 0.034 | -0.35, 0.42 | 0.86 |
| 6. isovaleric | -0.22 | -0.79, 0.34 | 0.44 |
| 7. caproic | 0.00 | -0.038, 0.038 | 0.99 |
| 8. isocaproic | -0.036 | -0.081, 0.01 | 0.12 |

* n infants=259

p-value (Pillai) adjusted for gestational age, c. section and cumulative breastfeeding.

# Supplementary Figure S1: Use of iron supplements by week of pregnancy and lowest haemoglobin measured during week 0-30.

# Supplementary Figure S2. Association between SNPs proximal to the hemochromatosis associated *HFE* rs1800462 and cord blood DNA methylation at the five CpG sites most significantly associated with this SNP in the MoBa cohort.

The three replicated differentially methylated CpG sites in the ALSPAC cohort were cg18357371 (near gene *SLC17A3*), cg07011110 (near gene *BTN1A1*) and cg13736514 (near gene *BTN3A2*), see Main figure 3.

In a) the x-axis shows distance to rs1800562 in kbp. The y-axis shows the negative logarithm of the p-value for differential methylation at each CpG site for the n SNPs proximal to rs1800562. The two SNPs in strong LD with rs1800562 are in red (rs144867591) and blue (rs79220007), see b) for details.

In b) the x-axis shows the linkage disequilibrium (LD) between rs1800562 and each of the proximal SNPs for which the –log(p) is indicated on the y-axis. The two SNPs in strong LD with rs1800462 are rs144867591 (-20149 bp from rs1800462, in red) and rs79220007 (5333 bp from rs1800462, in blue).

#


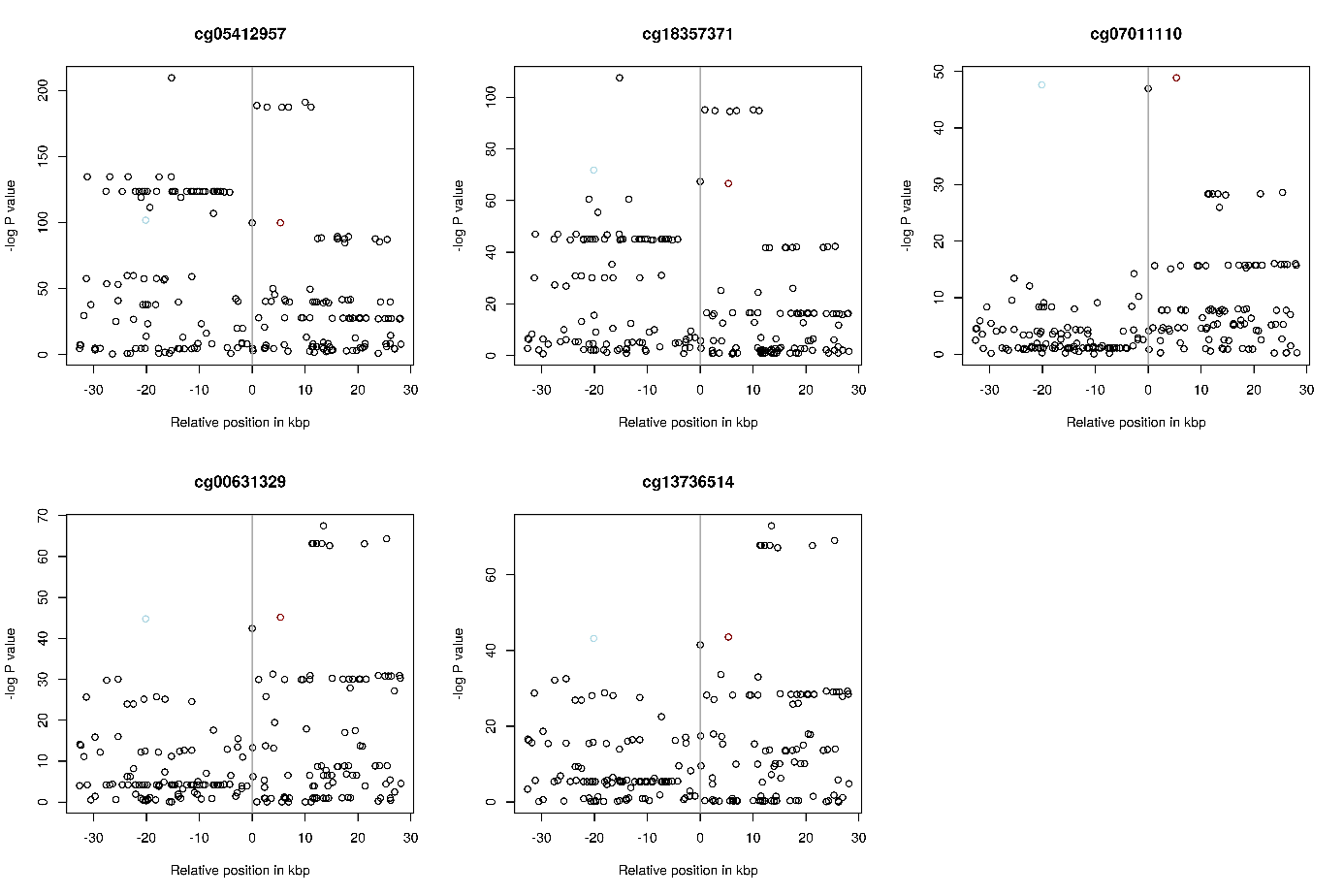


a)


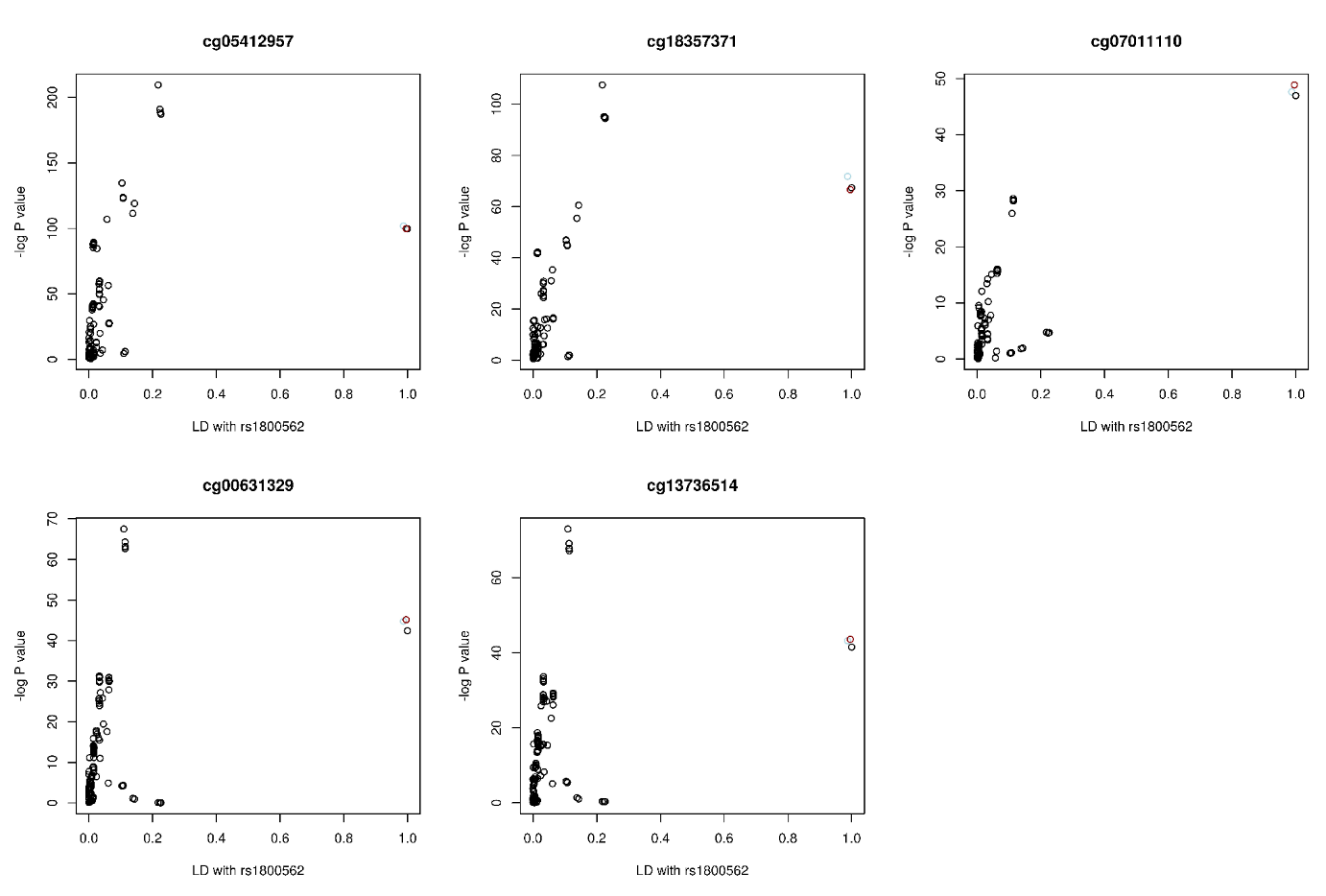


b)

# Supplementary Figure S3a-c: Post-hoc analysis of previously established non-HFE SNPs linked to iron status and genome wide cord blood DNA methylation in samples from the MoBa cohort.

Horizontal lines are for significance level (p=10^-8^), blue dots are significant CpGs.

1. rs3811647 in the *TF* gene (chromosome 3) encoding transferrin and shown to be associated with iron status (*12*).
2. rs855791 in the *TMPRSS6* gene (chromosome 22) encoding transmembrane protease, serine 6 and shown to be associated with iron status (*12*).
3. rs4820268 proximal to the *TMPRSS6* gene (chromosome 22) encoding transmembrane protease, serine 6 and shown to be associated with iron status (*12*).


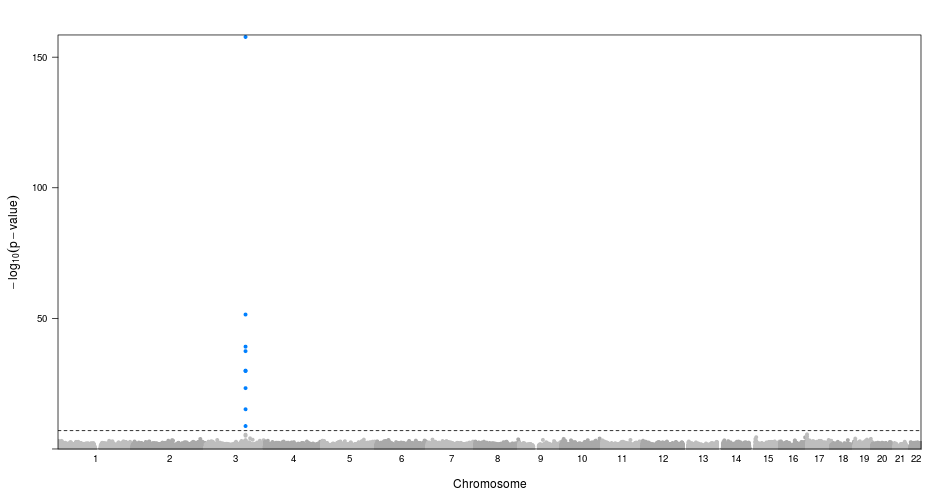


1.
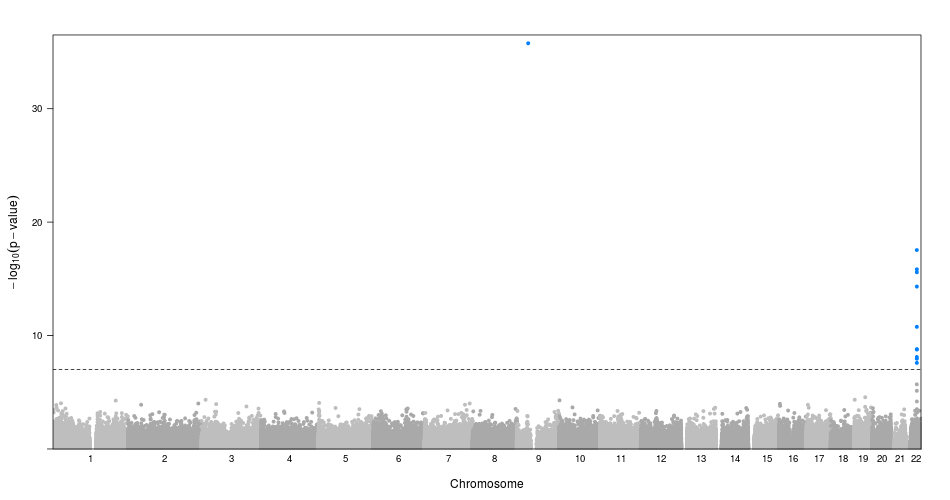


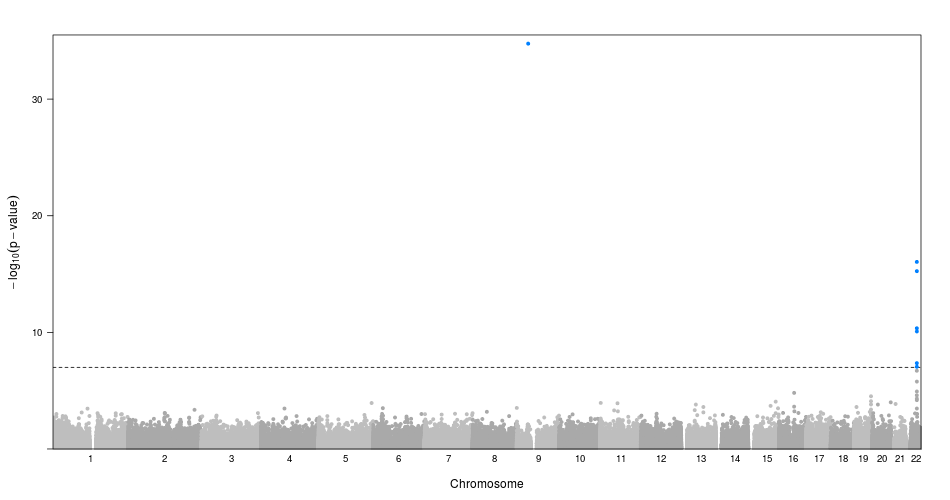


c)

# Supplementary Figure S4: Box-plot of cytokine z-scores (maternal samples approximately week 17 of pregnancy) by iron supplement use week 0-17 from randomly selected controls (n=519).

Panel A shows M1 immune markers (0=no iron supplements, 1=iron supplements used).

Panel B shows all the other measured immune markers

Panel C shows combined Z-scores for the groups of immune markers. The combined immune scores were generated by adding the individual z-scores for each immune marker in the group. P-values by LR-test was; for M1=0.04, M2=0.54, TH1=O.30, TH2=0.75, TH17=0.71, respectively.

Abbreviations: Interleukin (IL)-1β, IL-1 receptor antagonist (IL-1Ra), IL-2 receptor α (IL-2Ra), IL-2, -4, -5, -6, -10, -12p70, -13, -17A, granulocyte-macrophage colony stimulating factor (GM-CSF), interferon (IFN)-γ, C-X-C motif chemokine 10 (CXCL10, interferon gamma-induced protein 10), C-C motif chemokine ligand (CCL) 2 (monocyte chemo-attractant protein), CCL3 (macrophage inflammatory protein (MIP) 1α), CCL4 (MIP-1β), tumor necrosis factor (TNF)-α).


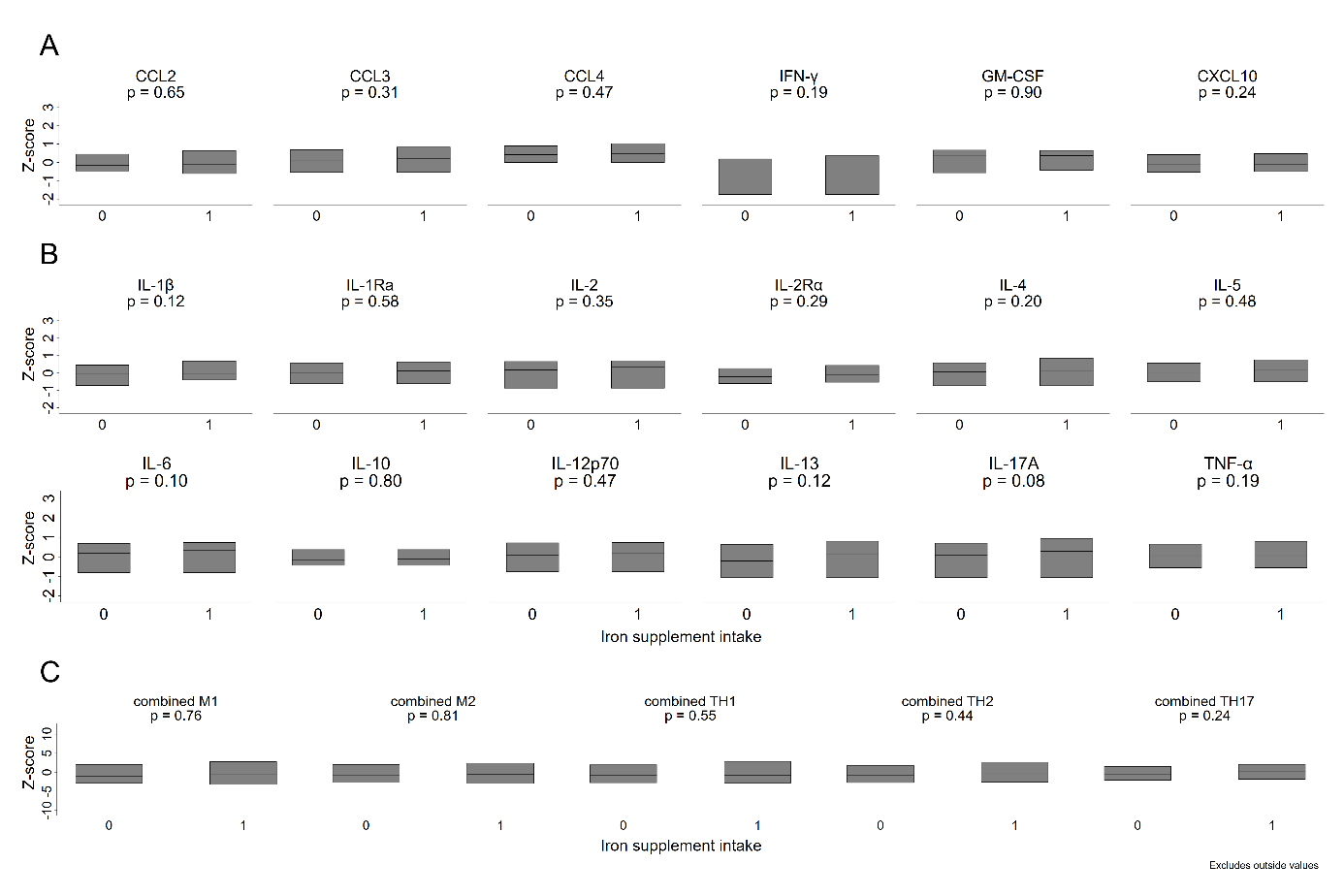


# Supplementary Figure S5: Concentrations of interferon (IFN)-γ and CCL4 in maternal mid-pregnancy samples, by maternal *HFE* (rs1800562) genotype.

0, 1 and 2 on the x-axis indicates the number of p.282Y alleles. The y-axis shows Z-score for each of the two cytokines, derived by subtracting the overall mean and dividing by the overall standard deviation such that zero corresponds to the average level.

FD: Fold difference

p-value: Test for trend per category change in number of p.282C alleles.

# Supplementary Figure S6. Abundance of bacterial families (Operational Taxonomic Units, OTU) in fecal samples from a) mothers at delivery b) infant at age 10 days and c) infant at age 30 days, by maternal use of iron supplements (Iron: No/Yes).

The Y-axis indicates the log of relative abundances of each detected OTU.

1. OTU194353: family Lachnospiraceae, genus Blautia

OTU185203: family Ruminococcaceae

OTU345176: family Ruminococcaceae

OTU190183: family Lachnospiraceae, genus Ruminococcus

1. OTU510870: family Enterobacteriaceae

OTU511908: family Enterobacteriaceae, genus Klebsiella

OTU528421: family Enterobacteriaceae

1. OTU3723096: Family Streptococcaceae

a)

b)

c)

# References

1 Mårild, K. *et al.* Midpregnancy and cord blood immunologic biomarkers, HLA genotype, and pediatric celiac disease. *J Allergy Clin Immunol*, doi:10.1016/j.jaci.2016.10.016 (2016).

2 Alexander, J. & Kowdley, K. V. HFE-associated hereditary hemochromatosis. *Genet Med* **11**, 307-313, doi:10.1097/GIM.0b013e31819d30f2 (2009).

3 Dilthey, A. *et al.* Multi-population classical HLA type imputation. *PLoS Comput Biol* **9**, e1002877, doi:10.1371/journal.pcbi.1002877 (2013).

4 Allen, K. J. *et al.* Iron-overload-related disease in HFE hereditary hemochromatosis. *N Engl J Med* **358**, 221-230, doi:10.1056/NEJMoa073286 (2008).

5 Genetics, D. o. C. E. *LD link*, <<https://analysistools.nci.nih.gov/LDlink/>>

6 Midttun, Ø., Hustad, S. & Ueland, P. M. Quantitative profiling of biomarkers related to B-vitamin status, tryptophan metabolism and inflammation in human plasma by liquid chromatography/tandem mass spectrometry. *Rapid communications in mass spectrometry : RCM* **23**, 1371-1379, doi:10.1002/rcm.4013 (2009).

7 Midttun, Ø. *et al.* Most blood biomarkers related to vitamin status, one-carbon metabolism, and the kynurenine pathway show adequate preanalytical stability and within-person reproducibility to allow assessment of exposure or nutritional status in healthy women and cardiovascular patients. *The Journal of nutrition* **144**, 784-790, doi:10.3945/jn.113.189738 (2014).

8 Joubert, B. R. *et al.* 450K epigenome-wide scan identifies differential DNA methylation in newborns related to maternal smoking during pregnancy. *Environ Health Perspect* **120**, 1425-1431, doi:10.1289/ehp.1205412 (2012).

9 Bibikova, M. *et al.* High density DNA methylation array with single CpG site resolution. *Genomics* **98**, 288-295, doi:10.1016/j.ygeno.2011.07.007 (2011).

10 Stanislawski, M. A. *et al.* Pre-pregnancy weight, gestational weight gain, and the gut microbiota of mothers and their infants. *Microbiome* **5**, 113, doi:10.1186/s40168-017-0332-0 (2017).

11 Dahl, C. *et al.* Preterm infants have distinct microbiomes, not explained by mode of delivery, less breastfeeding, or antibiotic exposure. *International journal of epidemiology*, In revision (2017).

12 Pichler, I. *et al.* Identification of a common variant in the TFR2 gene implicated in the physiological regulation of serum iron levels. *Hum Mol Genet* **20**, 1232-1240, doi:10.1093/hmg/ddq552 (2011).
